# First report of molecular epidemiology and phylogenetic characteristics of feline herpesvirus (FHV-1) from naturally infected cats in Kunshan, China

**DOI:** 10.1186/s12985-024-02391-1

**Published:** 2024-05-22

**Authors:** Semin Kim, Yixi Cheng, Zhenkun Fang, Qiu Zhongqi, Yu Weidong, Aysun Yilmaz, Huseyin Yilmaz, Sajid Umar

**Affiliations:** 1 Global Health Research Center (GHRC), Duke Kunshan University, No. 8 Duke Ave, 215316 Kunshan, China; 2 Simba Pet Hospital (Tinglin Park branch), Maanshan road, Kunshan, Suzhou, 215335 Jiangsu Province China; 3 Play Pi Kangkang Pet Hospital, Kunshan City Development Zone, Kunshan, Suzhou, 215300 Jiangsu Province China; 4 Department of Virology, Veterinary Faculty, Istanbul University-Cerrahpasa, Büyükcekmece, 35500 Istanbul, Türkiye; 5 Division of Natural & Applied Sciences (DNAS), Duke Kunshan University, Kunshan, China

**Keywords:** Feline herpesvirus, Epidemiology, Genetic diversity, Phylogenetic analysis, China

## Abstract

**Background:**

Feline herpesvirus type 1 (FHV-1) is a life threatening highly contagious virus in cats and typically causes upper respiratory tract infections as well as conjunctival and corneal ulcers. Genetic variability could alter the severity of diseases and clinical signs. Despite regular vaccine practices against FHV-1 in China, new FHV-1 cases still commonly occur. The genetic and phylogenetic characteristics of FHV-1 in Kunshan city of China has not been studied yet. Therefore, this study was planned to investigate the prevalence, molecular characteristics of circulating strains, and phylogenetic analyses of FHV-1. This is the first report of molecular epidemiology and phylogenetic characteristics of FHV-1 from naturally infected cats in Kunshan, China.

**Methods:**

The occulo-nasal swabs were collected from diseased cats showing respiratory distress, conjunctivitis, and corneal ulcers at different veterinary clinics in Kunshan from 2022 to 2023. Clinical data and general information were recorded. Swab samples were processed for preliminary detection of FHV-1. Thymidine kinase (*TK*), glycoprotein B (*gB*) and glycoprotein D (*gD*) genes were sequenced and analyzed to investigate genetic diversity and evolution of FHV-1.

**Results:**

The FHV-1 genome was detected in 43 (43/200, 21.5%) samples using RT-PCR targeting the TK gene. Statistical analysis showed a significant correlation between age, vaccination status and living environment (*p* < 0.05) with FHV-1 positivity, while a non-significant correlation was observed for FHV-1 positivity and sex of cats (*p* > 0.05). Additionally, eight FHV-1 positive cats were co-infected with feline calicivirus (8/43,18.6%). FHV-1 identified in the present study was confirmed as FHV-1 based on phylogenetic analyses. The sequence analyses revealed that 43 FHV-1 strains identified in the present study did not differ much with reference strains within China and worldwide. A nucleotide homology of 99-100% was determined among gB, TK and gD genes nucleotide sequences when compared with standard strain C-27 and vaccine strains. Amino acid analysis showed some amino acid substitutions in TK, gB and gD protein sequences. A potential N-linked glycosylation site was observed in all TK protein sequences. Phylogenetic analyses revealed minor variations and short evolutionary distance among FHV-1 strains detected in this study.

**Conclusions:**

Our findings indicate that genomes of 43 FHV-1 strains are highly homogenous and antigenically similar, and the degree of variation in major envelope proteins between strains is low. This study demonstrated some useful data about prevalence, genetic characteristics, and evolution of FHV-1 in Kunshan, which may aid in future vaccine development.

**Supplementary Information:**

The online version contains supplementary material available at 10.1186/s12985-024-02391-1.

## Introduction

Feline herpesvirus (FHV-1) is a life-threatening contagious virus in cats which causes feline viral rhinotracheitis. FHV-1 was first isolated in 1957 in the USA by Crandell and Maurer [1] from a cat showing respiratory signs. Feline viral rhinotracheitis is characterized by sticky discharge from eyes and nose along with respiratory distress, conjunctivitis, and corneal ulcers in cats. Serological analyses estimated that up to 97% cats show seropositivity against FHV-1 and more than 80% of infected cats remain persistently infected throughout lifespan [2, 3]. Latently infected cats become carriers of FHV-1 and shed virus intermittently during their life. Younger animals are more susceptible to FHV-1 infection. A core vaccine including FHV-1, feline calicivirus (FCV), and feline panleukopenia virus (FPV) is commercially available and administered worldwide [4, 5]. Vaccines usually provide good protection against FHV-1 infections; however, vaccines cannot prevent the development of carrier state and infection in cats [3, 6, 7].

FHV-1 is an enveloped DNA virus of Varicellovirus genus in *Herpesviridae* family. The whole genome of feline herpesvirus is approximately 135 kb, consisting of a unique long (UL) and unique short (US) gene sequences. US gene is flanked by a pair of identical inverted repeats, called terminal short repeats (TRs) and inverted short repeats (IRs) [8–11]. There is only one serotype for all FHV-1 isolates. There are 23 virion linked protein in FHV-1 along with 13 glycoproteins in virus envelop [9, 10]. Potential virulence factor which are attributed to FHV-1 include thymidine kinase (TK, UL23), glycoprotein E (gE, US8), glycoprotein C (gC, UL44) and serine/threonine protein kinase (US3) [12]. FHV-1 surface glycoprotein B (gB, UL27) assists in virus attachment on host cell receptors and induces high titers of virus neutralizing antibodies. Glycoprotein D (gD, US6) facilitate activation of gB and triggers virus entry process into host cells [13, 14].

Domestic cats act as primary host for FHV-1. However, FHV-1 natural infections have also been reported in several wild animals including leopards, cheetahs, jaguars, and margays [15–17]. FHV-1 transmission occurs through natural routes of nose, mouth, and conjunctiva. Direct contact with infected cats is considered a main route of FHV-1 transmission. In addition, FHV-1 can also be transmitted among susceptible cats through indirect routes such as contaminated food, water, bedding toys and cages. Kittens usually become infected after 6–9 weeks of birth with FHV-1 due to decline in maternal antibodies. Infected cats exhibit occulo-nasal discharge, fever, keratitis, and pneumonia [10, 18]. Cases of FHV-1 Infections in domestic cats have been reported throughout worldwide [6–8, 10, 19–26]. In China, several researchers have reported FHV-1 detection and isolation from Chengdu, Nanjing, Shanghai, Beijing, Hefei, Qingdao, and Harbin [23, 27–36]. However, detection rate of FHV-1 is variable worldwide including China that indicate many factors play a role in its epidemiology including housing, feeding, climate and vaccination. Molecular and serological assays are used to diagnosis of FHV-1 infection [10]. Vaccination immune responses and sero-epidemiological status of FHV-1 are monitored through virus neutralization assays [10, 37]. FHV-1 has become endemic and being detected regularly worldwide from cats and tigers [6, 10, 16]. At present, China ranks second after United states in the world in terms of the number of dogs and cats. In China, respiratory infections among cats are quite frequent and are usually caused by FHV-1 and FCV independently or in combinations.

There is scarcity of data on the epidemiology of cat infectious diseases particularly FHV-1 in China. Secondly, most of the data have been published in local journals in Chinese language which is not easily accessible outside China. The primary aims of this study was to investigate current status, molecular characteristics, and phylogenetic analysis of FHV-1 in China for better preventive measures and future vaccine development. This is the first report of molecular epidemiology and phylogenetic characteristics of FHV-1 from naturally infected cats in Kunshan, China.

## Material & methods

### Collection of samples

No ethical approval was required for this study. All procedures were performed following the international ethical standards for animal research. A total of 200 oculo-nasal specimens were collected from diseased cats showing respiratory distress, conjunctivitis, and corneal ulcers at different veterinary clinics in Kunshan from 2022 to 2023 (Supplementary Figure S1). Nasal and ocular swabs from a single cat were pooled into a tube containing 5 ml virus transport media (Copan Diagnostics Inc, Italy). General information about age, sex, breed, living, and vaccination status were recorded by clinic staff. The specimens were then transported in cold storage to Global Health Research Laboratory, Duke Kunshan University, China. All specimens were stored at -80 °C until analyzed.

### DNA Extraction and PCR screening for FHV-1

DNA was extracted from pooled swab samples by using a commercial DNA extraction (Takara, Dalian, China). DNA of FHV-1 was detected in the samples a conventional PCR assay to amplify a small segment of TK gene as described previously [6, 38]. Supplementary Table 1 lists the primers and thermocycling conditions for detection of FHV-1. PCR amplicons of approximately 287–293 bp were analyzed on 1.5% agarose gels.

### Sequencing PCR for full TK, gB and gD genes

Amplification of the full TK, gB and gD genes was achieved by using previously reported methods [39]. To validate PCR reaction, we added negative and positive controls in each PCR reaction. PCR amplicons of 531 bp, 1096 bp and 1125 bp were analyzed on 2% agarose gel for TK, gB and gD genes respectively by using gel imaging analysis system (Clinx, Shanghai, China). The positive PCR products were sequenced for genotypic and phylogenetic analysis. These sequences of TK, gB and gD genes were deposited in the GenBank (accession numbers OR504620-OR504748).

### Phylogenetic analyses

BioEdit Software (Ibis Biosciences, Carlsbad, CA, USA) was used to edit, align, and analyze nucleotide sequences of TK, gB and gD genes in the present study. Reference sequences including prototype C-27 FHV-1 strain for each gene were retrieved from NCBI GenBank database (http://www.ncbi.nlm.nih.gov). We blasted all sequences from present study for pairwise alignment and nucleotide homology comparison on NCBI GenBank database. Clustal W program was used for multiple sequence alignment in BioEdit. Nucleotide and amino acid comparisons were performed with reference sequences to understand the molecular epidemiology of identified FHV-1 in this study. The evolutionary history was inferred by using the Maximum Likelihood method and Tamura-Nei model (1,000 bootstrap replicates) [40]. The percentage of trees in which the associated taxa clustered together is shown next to the branches. The tree is drawn to scale, with branch lengths measured in the number of substitutions per site. Evolutionary analyses were conducted in MEGA11 [41].

### Statistical analysis

Statistical analysis was carried out among different parameters (age, sex, breed, living conditions, and vaccination status) using Pearson’s Chi-squared test (χ2) in *R* 4.1.0. A value of *p* < 0.05 and *p* < 0.01 were considered as statistically significant or highly significant.

## Results

### Detection of FHV-1 and clinical findings

FHV-1 was detected in 43 (43/200, 21.5%) swab samples by using PCR assays. A total of 157 samples were found negative for FHV-1(157/200, 78.5%) in this study. FHV-1 was detected among 12 (27.90%) vaccinated cats and 31 (72.09%) non-vaccinated cats. A total of 38 FHV-1 positive cats (88.37%) were below one year of age while 5 positive cats (11.6%) were aged above one year. Out of 43 positive cats, 26 (60.46%) were male while 17 (39.53%) (Table 1, supplementary Table S2). Among 89 cats living in groups, 27 tested positive, and among 111 cats living alone, 16 tested positive. The prominent clinical signs were sticky eyes and lacrimation along with respiratory distress, nasal discharge, conjunctivitis oral ulcers, anorexia, and fever. The sticky ocular discharge was more profuse and intense among non-vaccinated cats (personal observation). Statistical analysis showed a significant correlation between age, vaccination status and living environment (*p* < 0.05) with FHV-1 positivity, while a non-significant correlation was observed for FHV-1 positivity and sex of cats (*p* > 0.05). Additionally, eight FHV-1 positive cats were co-infected with feline calicivirus (8/43,18.6%). The severity of clinical signs was higher in coinfected cats than mono-infected cats.

**Table 1:** Statistical analysis of FHV-1 positive rate

Parameter	Total number of samples		FHV-1 positive number	Positive rate (%)	X^2^	*p*
	*n* = 200		43	21.5		
**Gender**						
Male	121		26	21.487	0.0000278	0.9958
Female	79		17	21.518		
**Age**						
≤ 1 year	113		38	33.628	22.64	< 0.0001
> 1 year	87		5	5.747		
**Residential density**						
Single	111		16	14.414	7.42	0.00645
Multiple	89		27	30.337		
**Vaccination status**						
Vaccinated	127		12	9.448	29.941	< 0.0001
Non-vaccinated	73		31	42.465		

### Nucleotide homology and phylogenetic analysis of FHV-1 genes

Pairwise comparative analysis showed a homology of 99–100% for nucleotide sequences between the 43 FHV-1 strains detected in the present study. The limited geographical range may have influenced this pattern. The sequence analyses revealed that 43 FHV-1 strains of this study did not differ much with reference strains within China and worldwide. A nucleotide homology of 99-100% was noticed among gB, TK and gD genes nucleotide sequences when compared with standard strain C-27 and vaccine strains. Non-synonymous nucleotide substitutions were observed in twenty-five TK gene sequences at two positions (T**78**A, C**184**G**)** while eighteen TK gene sequences did not show any substation. Non-synonymous nucleotide substitutions were noticed at some positions (C**59**T, T**60**A, G**1007**C, G**101**3T, A**67**T) within gB gene sequences. One synonymous nucleotide substitution (T**57**A) was observed in fourteen gB sequences which did not change amino acid constitution. Nucleotide analysis of gD gene revealed two synonymous substitutions (C143T, G**915**C) and one non-synonymous substitution (C**46**A). Amino acid analysis of TK, gB and gD protein sequences has been presented in additional files (additional file 1,2,3). Two amino acid substitutions (Asp**26**Glu, Ala**62**Gly) were observed in TK protein of several sequences in this study. Amino acid analysis of gB protein revealed some nonsynonymous amino acid substitutions (T20I, I23F, R336P, T338F). Interestingly, one sequence of gB protein in present study (OR504665) showed an amino acid substitution at position 20 (Thr**20**Ile) which was not noticed in all other sequences of present study. Only one nonsynonymous substitution (P16T) was observed in four gD protein sequences (OR504722, OR504723, OR504733, OR504734). A potential N-linked glycosylation site (N-X-S/T) was also observed at position 224 in all the sequences of TK protein.

**Fig. 1:**
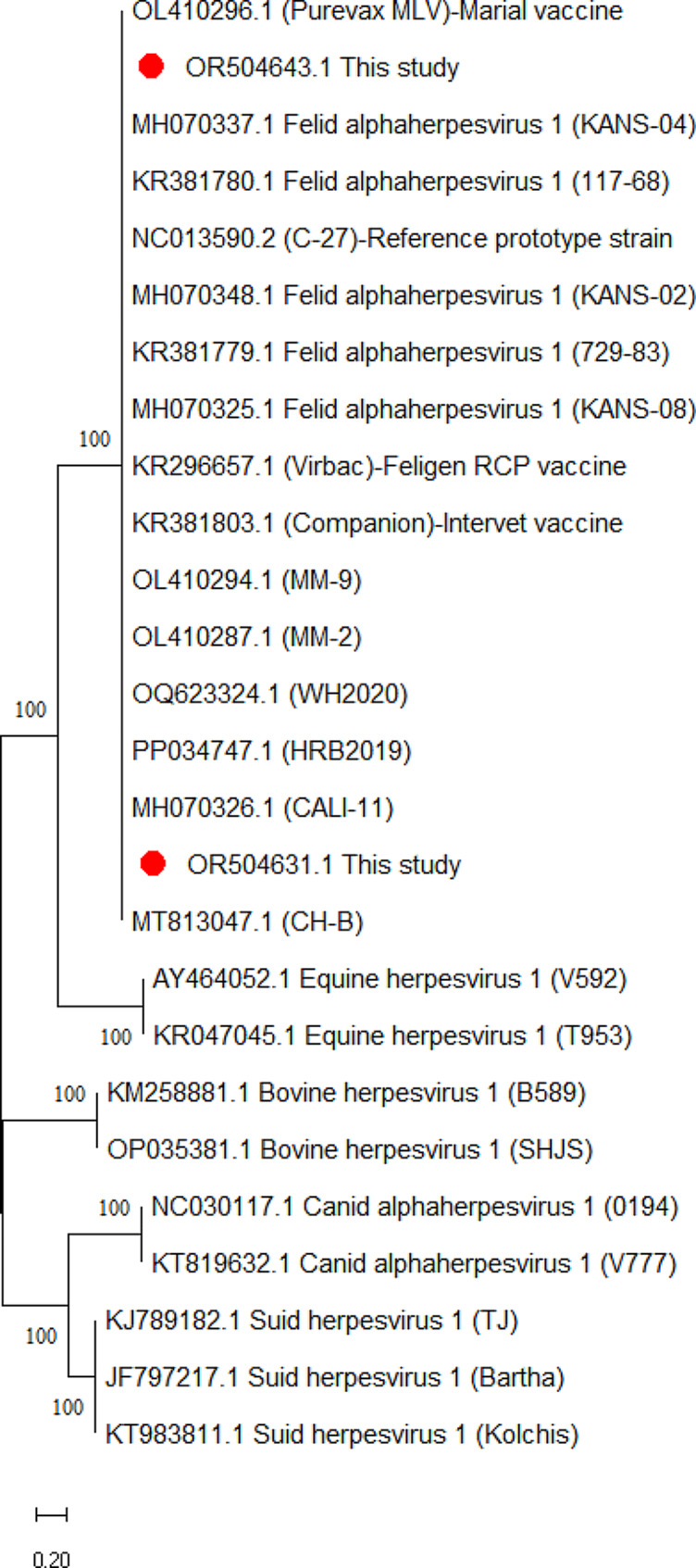
Phylogenetic trees based on the thymidine kinase (TK) gene sequences of FHV-1 strains detected in the present study and animal herpesvirus. Representative FHV-1 strains of present study (GenBank ID: OR504631, OR504643) are indicated with “red” circles. The scale bar indicates substitutions per amino acid residue

**Fig. 2:**
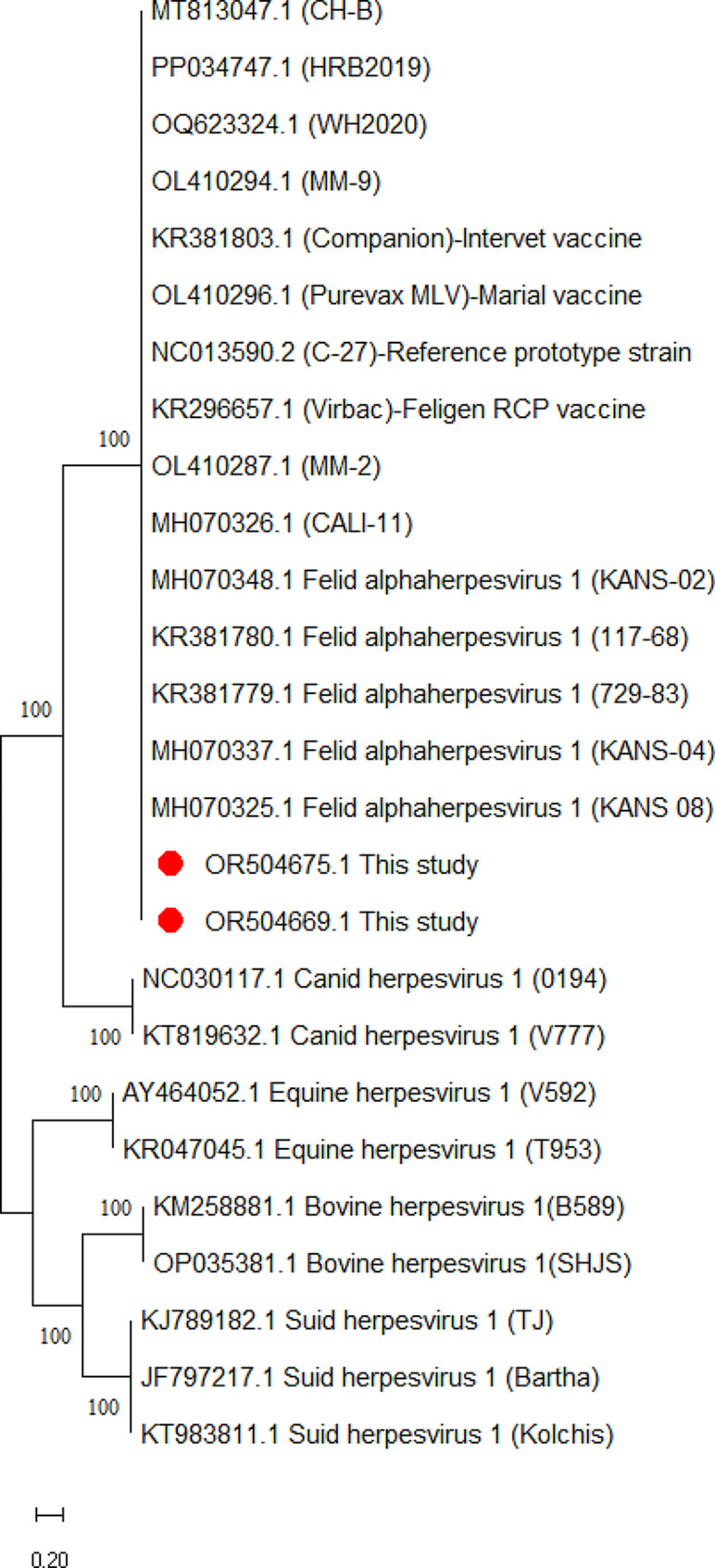
Phylogenetic trees based on the glycoprotein B (gB) gene sequences of FHV-1 strains detected in the present study and animal herpesvirus. Representative FHV-1 strains of present study (GenBank ID: OR504669, OR504675) are indicated with “red” circles. The scale bar indicates substitutions per amino acid residue

**Fig. 3:**
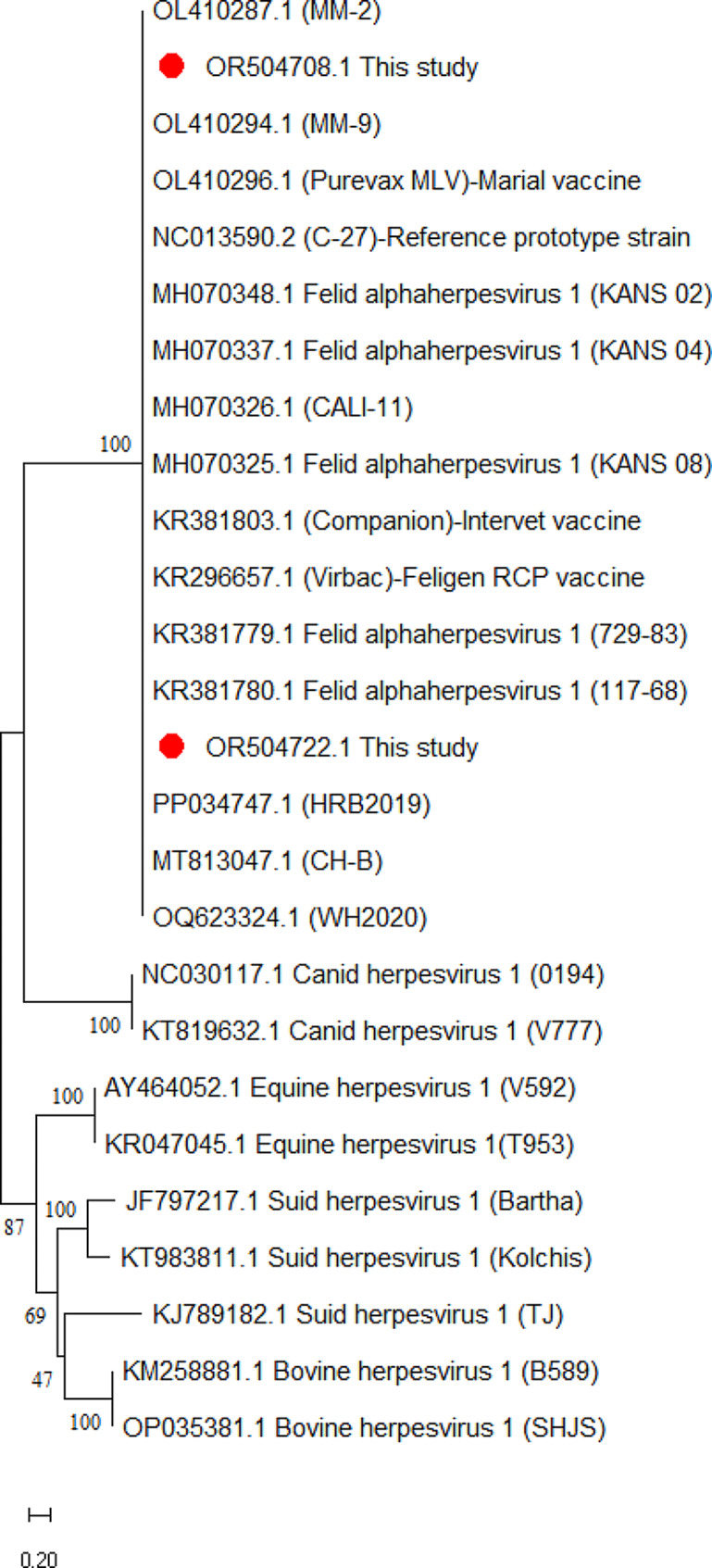
Phylogenetic trees based on the glycoprotein D (gD) gene sequences of FHV-1 strains detected in the present study and animal herpesvirus. Representative FHV-1 strains of present study (GenBank ID: OR504708, OR504722) are indicated with “red” circles. The scale bar indicates substitutions per amino acid residue

A phylogenetic tree was constructed to reveal the phylogenetic characteristics of 43 FHV-1 strains identified in this study. Only two representatives for each gene were selected for construction of a balanced phylogenetic tree. Phylogenetic analyses of TK, gB and gD gene sequences revealed high affinity and low evolutionary distance among strains. FHV-1 strains clustered closely with type 1 FHV-1 reference strains from China and USA. Representatives for each gene were denoted by red circles. However, FHV-1 strains detected in the present were distantly related to canine herpesvirus, equine herpesvirus, bovine herpesvirus, and swine herpesvirus strains (Figs. 1 and 2, 3).

## Discussion

Viral respiratory diseases are a serious health issue of cats worldwide [25, 42, 43]. Feline herpesvirus-1 (FHV-1), feline calicivirus (FCV), *Mycoplasma felis*, *Chlamydia felis* and *Bordetella bronchiseptica* are considered main etiological agents of feline upper respiratory tract infections [3, 5, 24, 25, 44]. FHV-1 is considered a main etiological agent for respiratory infections in cats and according to an estimate about 50–70% of cases of respiratory infections are associated with FHV-1 [45]. Therefore, a better understanding of epidemiological data, genetic characteristics is required for better preventive measures and future vaccine designs against FHV-1. There is a lack of data on regional epidemiology and phylogenetic characteristic of FHV-1 in China. This is first report to investigate the prevalence and phylogenetic characteristics of circulating FHV-1 strains in China.

During this study, 200 occulo-nasal swabs were collected from cats in Kunshan city in Jiangsu province of China and analyzed for the presence of FHV-1-DNA. FHV-1 detection rate was higher when compared with some previously published reports from China and other countries [23, 29, 31, 46]. However, in some studies, a higher FHV-1 detection rate was reported than found in the present study [19, 28, 33]. Chuchu et al. [35] tested 420 clinical samples from cats and found a detection rate of 26.3% for FHV-1 from 2016 to 2017. The positive detection rate in Beijing was 21.9% [32]. Liu et al. [23] reported a detection rate of 16.3% from 12 Chinese cities in between 2016 and 2019. The detection rate of FHV-1 was 57.8% in another study from Shanghai [28]. The positive detection rate in some areas of southwest China from 2020 to 2021 was 17.94% for FHV-1 [29]. During 2019–2021, Weijie et al. [31] tested 250 clinical samples from Qingdao and Beijing and found FHV detection rate of 20.8% (52/250). Kang et al. [47] collected eye and oropharyngeal swabs from 78 rescue cats in a shelter in South Korea in 2005 for RT-PCR testing and found 49 (63%) to be FHV-1 positive. These findings indicate that FHV-1 detection rate in China and other countries has no obvious direction. Notably, eight FHV-1 positive specimens (18.6%) were found co-infected with FCV. We did not test bacterial pathogens such as *Chlamydia felis*, *Bordetella bronchiseptica and Mycoplasma felis* which are known to exacerbate clinical signs and play a significant role in the development of feline respiratory disease complex. Co-infection with FHV-1, FCV and other respiratory pathogens have also been reported in other studies [6, 19, 23, 25, 29, 30, 33, 35]. Chuchu et al. [35], Liu et al. [23], Niu et al. [30] and Lijing et al. [29] reported a co-infection rates of 11.8%,1.04%, 8.33% and 10.14%, respectively between these two viruses. Sneezing, lacrimation, nose secretions, keratitis, cough, erosion of the nasal mucosa and corneal surface were main clinical signs among sick cats in the present study which is similar to those reported in previous studies [35, 42].

A total of 12 cats (27.90%) were found positive in this study despite being vaccinated against FHV-1. There are two possible explanations for this: (i) vaccinated cats can become virus carrier and spread infection horizontally to in contact animals if they encounter virus challenge (ii) kittens may get infection on maternal antibody decline prior to getting vaccines [24, 48]. Vaccines do not prevent FHV-1 infections although they can decrease the overall severity of the disease [6, 49]. Infected cats become persistent carriers due to FHV-1 latency [24, 50]. FHV-1 infection is prevalent in China despite continuous vaccination programs. One clinically licensed vaccine for immunization against FHV, FCV and feline parvovirus (core vaccine by Zoetis, USA) is being used in China which is recommended by the American Animal Hospital Association (AAHA) and the American Association of Feline Practitioners (AAFP). First dose (1 ml) of this vaccine is administer subcutaneously around 12 weeks of age or older. Healthy cats should receive 2 doses administered 3–4 weeks apart. Fully vaccinated cats are revaccinated after every one year. Based on personal communication with veterinarians in Kunshan city usually vaccines are administered on schedule with some exceptions. Sometime cat owners do not follow a vaccine schedule mainly due to lack of education or awareness. Young cats could get this virus from surroundings even after getting proper vaccines against FHV-1 and may become carriers of the virus in later stages. However, non-vaccinated cats are more prone to catch FHV-1 from environment. Additionally, clinical signs among vaccinated cats were generally mild than non-vaccinated cats. Previously, 44% of FHV-1 vaccinated cats sero-converted in a serological survey in China [23] which indicates that current commercial vaccines may not provide sufficient protection against the challenge of wild strains. In another study in Beijing, researchers reported high detection rate of FHV-1 in cats by using serological assays [51].

Statistical analysis revealed a significant association between FHV-1 positivity, multi-cat environment and age below one year which is consistent with previous studies [33, 35].These findings indicate that the FHV-1 infection rate is associated with the living environment and age of cats. However, a non-significant association was observed between breed of cats and FHV-1 detection rate in this study (*p* > 0.05). This present study categorized all purebred cats into one group. Thus, FHV-1 infection susceptibility for specific breeds cannot be determined in this study. This finding does not correlate with findings reported by Gao et al. [20] who reported higher incidence of FHV-1 in British Shorthair purebred cats. According to Tran et al. [52] purebred cats show more susceptibility for respiratory tract infections than mixed breeds. Perhaps, stress levels in purebred cats are high due to genetic differences [53]. The exact reason for this disparity is unknown and further investigations are needed [44]. The other factors such as environmental stress, living conditions and feeding status should also be investigated to reveal the exact story behind this disparity. There is also a higher risk of recurrence after stress or a change of environment. There were more male cases than female cases in the investigation, but with no significant difference in the proportion of confirmed cases which is in line with the previous findings from China [33]. However, this finding differs with the findings of Fernandez et al. [19] and Chuchu et al. [35] who reported no correlation between gender and viral infection. The difference could be due the fact that male cats are more aggressive and active than female cats and often spend more time outside their residence and thus leading to more chances of virus encounter [43, 54–56]. However, some researchers reported no difference in respiratory tract infections in male and female domestic cats [57]. In another study, FHV-1 infection rate in male cats were found significantly higher than female cats [20, 58].

We observed significantly higher detection rate of FHV-1 in younger cats than adults which is consistent with previous studies [19, 20, 56, 59] and disagreed with Chuchu et al. [35]. The most probable reasons for kittens susceptibility to FHV-1 infections could be the impaired or insufficient immunity [56]. Although older cats were tested negative for FHV1 infection, the result did not rule out latent infection. Even if the FHV1 PCR from nasal/ocular swab is negative, the cats with latent infection still have a chance to have reactivated disease and convert to be positive in the future.

Phylogenetic analysis revealed close association and clustering among FHV-1 strains detected in the present study and those retrieved from GenBank. The gB, gD and TK genes sequences revealed that FHV-1 strains are highly homogenous with domestic and foreign strains [31, 34]. Canine herpesvirus and FHV-1 strains of present study showed 67–74% similarity in nucleotide sequences. Interestingly, TK gene nucleotide sequences did not show much difference when compared with reference strains of FHV-1. The findings of the present study revealed that FHV-1 sequences did not have many variations and looked antigenically homogenous. Compared to other members of the Varicellovirus genus, FHV-1 has more conserved genome and variants are infrequent [7, 8, 10, 60]. Several sequences of TK, gB and gD proteins in this study were found to contain synonymous and non-synonymous amino acid substitutions. The pattern of amino acid substitutions in our study differed from previous studies from different geographical locations (USA, Australia, South Korea) of the World [7, 8, 10, 60]. Two unique amino acid substitutions (Asp**26**Glu, Ala**62**Gly) were observed in TK protein. Amino acid substitution (T**20**I) which was reported by Lewin et al. [8] previously, was not noticed in TK protein sequences of the present study. Similarly, amino acid substation (I**341**T) was also not observed in present study TK protein sequences which was reported by Yang et al. [10] in Korean FHV-1 isolates. These amino acid substitutions are in the region which are the main target of antiviral medications. There is a strong possibility of resistance against antiviral medications due to these SNPs [8]. There are some studies who have reported some non-synonymous mutations in other genes of FHV-1 including gB, gD, gC, gL, UL17 [24, 31]. Moreover, three unique nonsynonymous substitution were observed in gB (UL27) while no nonsynonymous substitution was observed in gD (US6) [8]. In another study, Lewin et al. [60] did not observe substitutions in TK (UL23) and DNA polymerase (UL30 and UL42) which are associated with virus replication. The amino acid substitution at position 341 of TK protein (I**341**T) were observed in Korean FHV-1 isolates [10]. Weijie et al. [31] reported two nonsynonymous mutation (M165) in amino acid sequence of gL gene while non synonymous mutation (A342T) in amino acid sequence of gD gene of FHV-1 strains isolated from Qingdao (Shandong) and Beijing, China during 2019–2021. Similar to a previous study [10], a potential N-linked glycosylation site (NTS) was in all TK protein sequence. This glycosylation site may have a dramatic impact of virus replication and transmissibility. The impact of these mutations on the function and structure of proteins is still not clear and needs further studies. This study has some limitations; First, we could not isolate FHV-1 and study bacterial pathogens in this study. Second, our study relied on pooled samples which cannot tell us about FHV-1 detection among individual samples from eyes and nose. Finally, we could not study recombination events among FHV-1 isolates and attenuated vaccine strains in this study which could have added an additional information for genotypic analysis.

In summary, our findings indicate that genomes of 43 FHV-1 strains are highly homogenous and antigenically similar, and the degree of variation in major envelope proteins between strains is low. This study demonstrated some useful data about prevalence, genetic characteristics, and evolution of FHV-1 in Kunshan, which may aid in future vaccine development.

### Electronic supplementary material

Below is the link to the electronic supplementary material.

**Supplementary Figure 1**. Map showing sampling area (Kunshan, China)

Supplementary Material 2

Supplementary Material 3

Supplementary Material 4

Supplementary Material 5

## Data Availability

No datasets were generated or analysed during the current study.

## References

[1] Crandell RA, Maurer FD (1958). Isolation of a feline virus associated with intranuclear inclusion bodies. Proc Soc Exp Biol Med.

[2] Maggs DJ, Clarke HE (2005). Relative sensitivity of polymerase chain reaction assays used for detection of feline herpesvirus type 1 DNA in clinical samples and commercial vaccines. Am J Vet Res.

[3] Cavalheiro JB, Echeverria JT, Ramos CAN, Babo-Terra VJ (2023). Frequency of feline herpesvirus 1 (FHV-1) in domestic cats from Campo Grande, MS, Brazil. Acad Bras Cienc.

[4] Reagan KL, Hawley JR, Lappin MR (2014). Concurrent administration of an intranasal vaccine containing feline herpesvirus-1 (FHV-1) with a parenteral vaccine containing FHV-1 is superior to parenteral vaccination alone in an acute FHV-1 challenge model. Vet J.

[5] Kim S, Cheng Y, Fang Z, Liu X, Zhongqi Q, Weidong Y, Yilmaz A, Yilmaz H, Umar S (2024). Molecular epidemiology and phylogenetic analysis of feline calicivirus in Kunshan, China. Virol J.

[6] Henzel A, Brum MC, Lautert C, Martins M, Lovato LT, Weiblen R (2012). Isolation and identification of feline calicivirus and feline herpesvirus in Southern Brazil. Braz J Microbiol.

[7] Vaz PK, Job N, Horsington J, Ficorilli N, Studdert MJ, Hartley CA, Gilkerson JR, Browning GF, Devlin JM (2016). Low genetic diversity among historical and contemporary clinical isolates of felid herpesvirus 1. BMC Genomics.

[8] Lewin AC, Kolb AW, McLellan GJ, Bentley E, Bernard KA, Newbury SP, Brandt CR (2018). Genomic, Recombinational and Phylogenetic Characterization of Global Feline Herpesvirus 1 isolates. Virology.

[9] Tai SH, Niikura M, Cheng HH, Kruger JM, Wise AG, Maes RK (2010). Complete genomic sequence and an infectious BAC clone of feline herpesvirus-1 (FHV-1). Virology.

[10] Yang D-K, Kim H-H, Park Y-R, Yoo JY, Choi S-S, Park Y, An S, Park J, Kim J, Kim H-J (2020). Isolation and Molecular Characterization of Feline Herpesvirus 1 from naturally infected Korean cats. J Bacteriol Virol.

[11] Tang A, Zhu M, Zhu J, Zhang D, Zhu S, Wang X, Meng C, Li C, Liu G (2023). Pathogenicity and immunogenicity of gI/gE/TK-gene-deleted Felid herpesvirus 1 variants in cats. Virol J.

[12] Gaskell R, Dawson S, Radford A, Thiry E (2007). Feline herpesvirus. Vet Res.

[13] Tan Y, Dong G, Xu H, Niu J, Lu W, Wang K, Dong H, Zhang S, Huang H, Hu G (2020). Development of a cross-priming isothermal amplification assay based on the glycoprotein B gene for instant and rapid detection of feline herpesvirus type 1. Arch Virol.

[14] Synowiec A, Dąbrowska A, Pachota M, Baouche M, Owczarek K, Niżański W, Pyrc K (2023). Feline herpesvirus 1 (FHV-1) enters the cell by receptor-mediated endocytosis. J Virol.

[15] Shi L, Huang S, Lu Y, Su Y, Guo L, Guo L, Xie W, Li X, Wang Y, Yang S (2022). Cross-species transmission of feline herpesvirus 1 (FHV-1) to chinchillas. Vet Med Sci.

[16] Sun H, Li Y, Jiao W, Liu C, Liu X, Wang H, Hua F, Dong J, Fan S, Yu Z (2014). Isolation and identification of feline herpesvirus type 1 from a South China tiger in China. Viruses.

[17] Xiao JX, Shan F, Huang JX, Chen W (2013). Diagnosis of rhinotracheitisvirus from siberian tiger (Panthera tigris altaica) and South China tiger (Panthera tigris amoyensis) by PCR and sequence analysis. Chin J Wildl.

[18] Lee Y, Maes R, Tai SS, Soboll Hussey G (2019). Viral replication and innate immunity of feline herpesvirus-1 virulence-associated genes in feline respiratory epithelial cells. Virus Res.

[19] Fernandez M, Manzanilla EG, Lloret A, León M, Thibault JC (2017). Prevalence of feline herpesvirus-1, feline calicivirus, Chlamydophila felis and Mycoplasma felis DNA and associated risk factors in cats in Spain with upper respiratory tract disease, conjunctivitis and/or gingivostomatitis. J Feline Med Surg.

[20] Gao J, Li Y, Xie Q, Al-Zaban MI, Al-Saeed FA, Shati AA, Al-Doaiss AA, Ahmed AE, Nawaz S, Ebrahem H et al. Epidemiological Investigation of Feline Upper Respiratory Tract Infection encourages a geographically specific FCV Vaccine. Vet Sci 2023, 10. 10.3390/vetsci10010046 PMC9864582 36669047

[21] Hora AS, Tonietti PO, Guerra JM, Leme MC, Pena HF, Maiorka PC, Brandão PE (2013). Felid herpesvirus 1 as a causative agent of severe nonsuppurative meningoencephalitis in a domestic cat. J Clin Microbiol.

[22] Johansson Ö, Ullman K, Lkhagvajav P, Wiseman M, Malmsten J, Leijon M. Detection and genetic characterization of viruses Present in Free-ranging Snow leopards using next-generation sequencing. Front Veterinary Sci 2020, 7. 10.3389/fvets.2020.00645 PMC7536260 33195503

[23] Liu C, Liu Y, Qian P, Cao Y, Wang J, Sun C, Huang B, Cui N, Huo N, Wu H (2020). Molecular and serological investigation of cat viral infectious diseases in China from 2016 to 2019. Transbound Emerg Dis.

[24] Magouz A, Lokman MS, Albrakati A, Elmahallawy EK. First report of isolation and molecular characterization of Felid Herpesvirus-1 from symptomatic domestic cats in Egypt. Vet Sci 2022, 9. 10.3390/vetsci9020081 PMC8874770 35202334

[25] Nguyen D, Barrs VR, Kelman M, Ward MP (2019). Feline upper respiratory tract infection and disease in Australia. J Feline Med Surg.

[26] Walter J, Foley P, Yason C, Vanderstichel R, Muckle A (2020). Prevalence of feline herpesvirus-1, feline calicivirus, Chlamydia felis, and Bordetella bronchiseptica in a population of shelter cats on Prince Edward Island. Can J Vet Res.

[27] Ji W. Isolation and identification of feline herpesvirus 1 in Beijing, China. *Animal Husbandry & Veterinary Medicine 34: 744–754 (in Chinese)* 2018.

[28] Huahua X. Epidemiological investigation and treatment effect evaluation of feline herpesvirus and calicivirus infection cases in Shanghai. *Yinchuan: Ningxia University, 2021 (in Chinese)* 2021.

[29] Lijing L. Molecular epidemiological investigation of feline herpesvirus type I and feline calicivirus infections in some areas of southwest China. Chengdu. *Southwest University for Nationalities (in Chinese)* 2021.

[30] Niu HZ, Ma XY. QB. Investigation and statistical analysis of 2249 cases of feline diseases in a pet hospital in Nanjing from 2018 to 2019.: *Animal Husbandry & Veterinary Medicine*, 2021,*53 (3):115–120 (in Chinese)*.

[31] Weijie (2023). Isolation, Identification and Biological characteristics of Feline Herpesvirus-1. Chin J Virol.

[32] Siyan (2018). Investigation on the main pathogens of 306 cases of cat upper respiratory tract infection in Beijing. Chin Veterinary J.

[33] Yuehan. Epidemiological investigation and analysis of feline herpesvirus type I and feline calicivirus in Anhui Province. *Hefei: Anhui Agricultural University (in chinese)* 2020.

[34] Xu Xinyan ZY, Liu D, Bo M, Jiasen L. Qu Liandong Isolation and Identification of a Feline Herpesvirus-1 Strain. *Acta Veterinaria et Zootechnica Sinica*, 2023, 54(4): 1713–1720 (in Chinese) 2023.

[35] Chuchu (2017). Epidemiologicin vestigate of feline FHV and FCV infection in Beijing. Chin J Veterinary Med.

[36] Wu. Isolation, identification, and whole-genome sequencing of FHV-1 in Guangzhou from 2016 to 2017. Guangzhou. *South China Agricultural University, 2018 (in Chinese)* 2018.

[37] Yagami K, Furukawa T, Fukui M (1985). Serologic and virologic surveys on feline herpesvirus and feline calicivirus infections in cats for experimental use. Jikken Dobutsu.

[38] Sykes JE, Allen JL, Studdert VP, Browning GF (2001). Detection of feline calicivirus, feline herpesvirus 1 and Chlamydia psittaci mucosal swabs by multiplex RT-PCR/PCR. Vet Microbiol.

[39] Wang Ji FR, Feng Yufang L, Xiaobo W, Shujing W, Shasha L, Wei (2018). Qin Xiao,Gong Wei, Yue Bingfei, he Zhengming: isolation and identification of feline herpesvirus type I in Beijing area. Chin J Virol.

[40] Tamura K, Nei M (1993). Estimation of the number of nucleotide substitutions in the control region of mitochondrial DNA in humans and chimpanzees. Mol Biol Evol.

[41] Tamura K, Stecher G, Kumar S (2021). MEGA11: Molecular Evolutionary Genetics Analysis Version 11. Mol Biol Evol.

[42] Cohn LA (2011). Feline respiratory disease complex. Vet Clin North Am Small Anim Pract.

[43] Wong WT, Kelman M, Ward MP (2013). Surveillance of upper respiratory tract disease in owned cats in Australia, 2009–2012. Prev Vet Med.

[44] Chan I, Dowsey A, Lait P, Tasker S, Blackwell E, Helps CR, Barker EN (2023). Prevalence and risk factors for common respiratory pathogens within a cohort of pet cats in the UK. J Small Anim Pract.

[45] Maes R. Felid herpesvirus type 1 infection in cats: a natural host model for alphaherpesvirus pathogenesis. *ISRN Vet Sci* 2012, 2012:495830. 10.5402/2012/495830 PMC3671728 23762586

[46] Berger A, Willi B, Meli ML, Boretti FS, Hartnack S, Dreyfus A, Lutz H, Hofmann-Lehmann R (2015). Feline calicivirus and other respiratory pathogens in cats with feline calicivirus-related symptoms and in clinically healthy cats in Switzerland. BMC Vet Res.

[47] Kang BT, Park HM (2008). Prevalence of feline herpesvirus 1, feline calicivirus and Chlamydophila felis in clinically normal cats at a Korean animal shelter. J Vet Sci.

[48] Holst BS, Berndtsson LT, Englund L (2005). Isolation of feline herpesvirus-1 and feline calicivirus from healthy cats in Swedish breeding catteries. J Feline Med Surg.

[49] Bergmann M, Speck S, Rieger A, Truyen U, Hartmann K (2020). Antibody response to feline herpesvirus-1 vaccination in healthy adult cats. J Feline Med Surg.

[50] Lee Y, Maes RK, Kruger JM, Kiupel M, Giessler KS, Soboll Hussey G. Safety and Efficacy of Felid Herpesvirus-1 deletion mutants in cats. Viruses 2021, 13. 10.3390/v13020163 PMC7911815 33499363

[51] Wang. Establishment and preliminary application of ELISA for detecting antibody to Felid Herpesvirus 1 in cat. Lab Anim Sci 2014.

[52] Tran V, Kelman M, Ward M, Westman M (2019). Risk of Feline Immunodeficiency Virus (FIV) infection in Pet cats in Australia is higher in Areas of Lower Socioeconomic Status. Animals.

[53] Sandøe P, Nørspang A, Forkman B, Bjornvad C, Kondrup SV, Lund T (2017). The burden of domestication: a representative study of welfare in privately owned cats in Denmark. Anim Welf.

[54] Loyd KA, Hernandez SM, Abernathy KJ, Shock BC, Marshall GJ (2013). Risk behaviours exhibited by free-roaming cats in a suburban US town. Vet Rec.

[55] Strickler BL, Shull EA (2014). An owner survey of toys, activities, and behavior problems in indoor cats. J Veterinary Behav.

[56] Dinnage JD, Scarlett JM, Richards JR (2009). Descriptive epidemiology of feline upper respiratory tract disease in an animal shelter. J Feline Med Surg.

[57] Bannasch MJ, Foley JE (2005). Epidemiologic evaluation of multiple respiratory pathogens in cats in animal shelters. J Feline Med Surg.

[58] Binns SH, Dawson S, Speakman AJ, Cuevas LE, Hart CA, Gaskell CJ, Morgan KL, Gaskell RM (2000). A study of feline upper respiratory tract disease with reference to prevalence and risk factors for infection with feline calicivirus and feline herpesvirus. J Feline Med Surg.

[59] Sykes JE, Anderson GA, Studdert VP, Browning GF (1999). Prevalence of feline Chlamydia psittaci and feline herpesvirus 1 in cats with upper respiratory tract disease. J Vet Intern Med.

[60] Lewin AC, Ineck NE, Mironovich MA, Marino ME, Liu CC, Emelogu U, Mills EP, Camacho-Luna P, Carter RT (2023). Surveillance for feline herpesvirus type 1 mutation and development of resistance in cats treated with antiviral medications. Front Vet Sci.

