# Supplementary figures and images for: First report of molecular epidemiology and phylogenetic characteristics of feline herpesvirus (FHV-1) from naturally infected cats in Kunshan, China
Source: Virol J. 2024 May 22;21:115. doi: 10.1186/s12985-024-02391-1 (PMC11112849; doi:10.1186/s12985-024-02391-1)

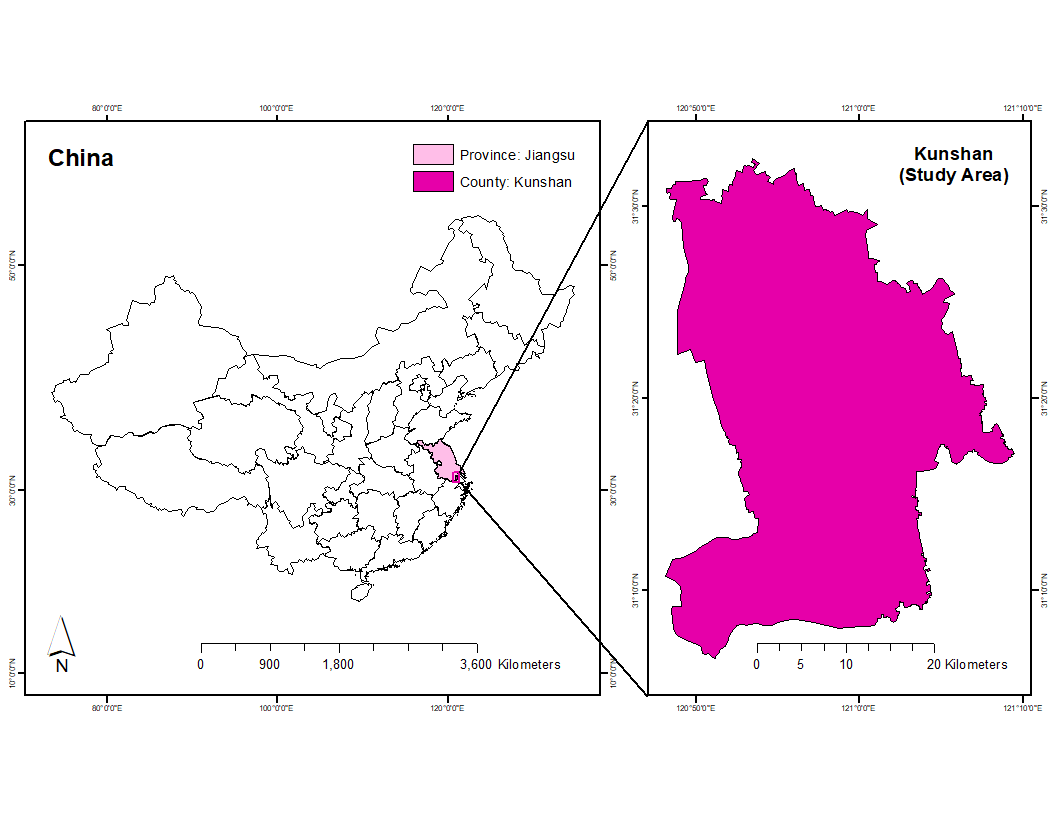

Supplement: Supplementary file 1 — Supplementary Figure 1. Map showing sampling area (Kunshan, China) [file 12985_2024_2391_MOESM1_ESM.png]
