# Supplementary material for: First report of molecular epidemiology and phylogenetic characteristics of feline herpesvirus (FHV-1) from naturally infected cats in Kunshan, China
Source: Virol J. 2024 May 22;21:115. doi: 10.1186/s12985-024-02391-1 (PMC11112849; doi:10.1186/s12985-024-02391-1)
Supplement: Supplementary file 2 — Supplementary Material 2 [file 12985_2024_2391_MOESM2_ESM.pdf]

```
                                    10         20         30         40         50         60         70         80         90
                              ....|....| ....|....| ....|....| ....|....| ....|....| ....|....| ....|....| ....|....| ....|....|
NC013590.2 (C-27)-Reference   MMTRLHFWWC GIXCGPEISG MYFKPYDHAK NNYGLCE--X DLIX---XST TLQLYSSQX- XRAKNSPGDG --SEDNS*ST LCNINGFMWD
OL410296.1 Marial vaccine     .......... .......... .......... .......--. ....---... .........- .......... --.....*.. ..........
KR296657.1 Virbac vaccine     .......... .......... .......... .......--. ....---... .........- .......... --.....*.. ..........
KR381803.1 Intervet vaccine   .......... .......... .......... .......--. ....---... .........- .......... --.....*.. ..........
MH070348.1(KANS-02)           .......... .......... .......... .......--. ....---... .........- .......... --.....*.. ..........
OR504706.1 This study         .......... .......... .......... .......--. ....---... .........- .......... --.....*.. ..........
OR504707.1 This study         .......... .......... .......... .......--. ....---... .........- .......... --.....*.. ..........
OR504708.1 This study         .......... .......... .......... .......--. ....---... .........- .......... --.....*.. ..........
OR504709.1 This study         .......... .......... .......... .......--. ....---... .........- .......... --.....*.. ..........
OR504710.1 This study         .......... .......... .......... .......--. ....---... .........- .......... --.....*.. ..........
OR504711.1 This study         .......... .......... .......... .......--. ....---... .........- .......... --.....*.. ..........
OR504712.1 This study         .......... .......... .......... .......--. ....---... .........- .......... --.....*.. ..........
OR504713.1 This study         .......... .......... .......... .......--. ....---... .........- .......... --.....*.. ..........
OR504714.1 This study         .......... .......... .......... .......--. ....---... .........- .......... --.....*.. ..........
OR504715.1 This study         .......... .......... .......... .......--. ....---... .........- .......... --.....*.. ..........
OR504716.1 This study         .......... .......... .......... .......--. ....---... .........- .......... --.....*.. ..........
```

```
                                    10         20         30         40         50         60         70         80         90
                              ....|....| ....|....| ....|....| ....|....| ....|....| ....|....| ....|....| ....|....| ....|....|
NC013590.2 (C-27)-Reference   MMTRLHFWWC GIXCGPEISG MYFKPYDHAK NNYGLCE--X DLIX---XST TLQLYSSQX- XRAKNSPGDG --SEDNS*ST LCNINGFMWD
OL410296.1 Marial vaccine     .......... .......... .......... .......--. ....---... .........- .......... --.....*.. ..........
KR296657.1 Virbac vaccine     .......... .......... .......... .......--. ....---... .........- .......... --.....*.. ..........
KR381803.1 Intervet vaccine   .......... .......... .......... .......--. ....---... .........- .......... --.....*.. ..........
MH070348.1(KANS-02)           .......... .......... .......... .......--. ....---... .........- .......... --.....*.. ..........
OR504717.1 This study         .......... .......... .......... .......--. ....---... .........- .......... --.....*.. ..........
OR504718.1 This study         .......... .......... .......... .......--. ....---... .........- .......... --.....*.. ..........
OR504719.1 This study         .......... .......... .......... .......--. ....---... .........- .......... --.....*.. ..........
OR504720.1 This study         .......... .......... .......... .......--. ....---... .........- .......... --.....*.. ..........
OR504721.1 This study         .......... .......... .......... .......--. ....---... .........- .......... --.....*.. ..........
OR504722.1 This study         .......... .....T.... .......... .......--. ....---... .........- .......... --.....*.. ..........
OR504723.1 This study         .......... .....T.... .......... .......--. ....---... .........- .......... --.....*.. ..........
OR504724.1 This study         .......... .......... .......... .......--. ....---... .........- .......... --.....*.. ..........
OR504725.1 This study         .......... .......... .......... .......--. ....---... .........- .......... --.....*.. ..........
OR504726.1 This study         .......... .......... .......... .......--. ....---... .........- .......... --.....*.. ..........
OR504727.1 This study         .......... .......... .......... .......--. ....---... .........- .......... --.....*.. ..........
```

```
                                         10         20         30         40         50         60         70         80         90
                                ....|....| ....|....| ....|....| ....|....| ....|....| ....|....| ....|....| ....|....| ....|....|
NC013590.2 (C-27)-Reference     MMTRLHFWWC GIXCGPEISG MYFKPYDHAK NNYGLCE--X DLIX---XST TLQLYSSQX- XRAKNSPGDG --SEDNS*ST LCNINGFMWD
OL410296.1 Marial vaccine       .......... .......... .......... .......--. ....---... .........- .......... --.....*.. ..........
KR296657.1 Virbac vaccine       .......... .......... .......... .......--. ....---... .........- .......... --.....*.. ..........
KR381803.1 Intervet vaccine     .......... .......... .......... .......--. ....---... .........- .......... --.....*.. ..........
MH070348.1(KANS-02)             .......... .......... .......... .......--. ....---... .........- .......... --.....*.. ..........
OR504728.1 This study           .......... .......... .......... .......--. ....---... .........- .......... --.....*.. ..........
OR504729.1 This study           .......... .......... .......... .......--. ....---... .........- .......... --.....*.. ..........
OR504730.1 This study           .......... .......... .......... .......--. ....---... .........- .......... --.....*.. ..........
OR504731.1 This study           .......... .......... .......... .......--. ....---... .........- .......... --.....*.. ..........
OR504732.1 This study           .......... .......... .......... .......--. ....---... .........- .......... --.....*.. ..........
OR504733.1 This study           .......... .....T.... .......... .......--. ....---... .........- .......... --.....*.. ..........
OR504734.1 This study           .......... .....T.... .......... .......--. ....---... .........- .......... --.....*.. ..........
OR504735.1 This study           .......... .......... .......... .......--. ....---... .........- .......... --.....*.. ..........
OR504736.1 This study           .......... .......... .......... .......--. ....---... .........- .......... --.....*.. ..........
OR504737.1 This study           .......... .......... .......... .......--. ....---... .........- .......... --.....*.. ..........
OR504738.1 This study           .......... .......... .......... .......--. ....---... .........- .......... --.....*.. ..........
```

```
                                         10         20         30         40         50         60         70         80         90
                                ....|....| ....|....| ....|....| ....|....| ....|....| ....|....| ....|....| ....|....| ....|....|
NC013590.2 (C-27)-Reference     MMTRLHFWWC GIXCGPEISG MYFKPYDHAK NNYGLCE--X DLIX---XST TLQLYSSQX- XRAKNSPGDG --SEDNS*ST LCNINGFMWD
OL410296.1 Marial vaccine       .......... .......... .......... .......--. ....---... .........- .......... --.....*.. ..........
KR296657.1 Virbac vaccine       .......... .......... .......... .......--. ....---... .........- .......... --.....*.. ..........
KR381803.1 Intervet vaccine     .......... .......... .......... .......--. ....---... .........- .......... --.....*.. ..........
MH070348.1(KANS-02)             .......... .......... .......... .......--. ....---... .........- .......... --.....*.. ..........
OR504739.1 This study           .......... .......... .......... .......--. ....---... .........- .......... --.....*.. ..........
OR504740.1 This study           .......... .......... .......... .......--. ....---... .........- .......... --.....*.. ..........
OR504741.1 This study           .......... .......... .......... .......--. ....---... .........- .......... --.....*.. ..........
OR504742.1 This study           .......... .......... .......... .......--. ....---... .........- .......... --.....*.. ..........
OR504743.1 This study           .......... .......... .......... .......--. ....---... .........- .......... --.....*.. ..........
OR504744.1 This study           .......... .......... .......... .......--. ....---... .........- .......... --.....*.. ..........
OR504745.1 This study           .......... .......... .......... .......--. ....---... .........- .......... --.....*.. ..........
OR504746.1 This study           .......... .......... .......... .......--. ....---... .........- .......... --.....*.. ..........
OR504747.1 This study           .......... .......... .......... .......--. ....---... .........- .......... --.....*.. ..........
OR504748.1 This study           .......... .......... .......... .......--. ....---... .........- .......... --.....*.. ..........
```

```
                                         100        110        120        130        140        150        160        170        180
                                 ....|....| ....|....| ....|....| ....|....| ....|....| ....|....| ....|....| ....|....| ....|....|
NC013590.2 (C-27)-Reference      GGIDIRAGYR RYYSNHTTIS KKNXX*RDYK LV*GNPGL*I PYVSYXI*DF VILNGNLEYV LYGRLHIGWN L*QSICS*XD DELGLIMMAP
OL410296.1 Marial vaccine        .......... .......... .....*.... ..*.....*. .......*.. .......... .......... .*.....*.. ..........
KR296657.1 Virbac vaccine        .......... .......... .....*.... ..*.....*. .......*.. .......... .......... .*.....*.. ..........
KR381803.1 Intervet vaccine      .......... .......... .....*.... ..*.....*. .......*.. .......... .......... .*.....*.. ..........
MH070348.1(KANS-02)              .......... .......... .....*.... ..*.....*. .......*.. .......... .......... .*.....*.. ..........
OR504706.1 This study            .......... .......... .....*.... ..*.....*. .......*.. .......... .......... .*.....*.. ..........
OR504707.1 This study            .......... .......... .....*.... ..*.....*. .......*.. .......... .......... .*.....*.. ..........
OR504708.1 This study            .......... .......... .....*.... ..*.....*. .......*.. .......... .......... .*.....*.. ..........
OR504709.1 This study            .......... .......... .....*.... ..*.....*. .......*.. .......... .......... .*.....*.. ..........
OR504710.1 This study            .......... .......... .....*.... ..*.....*. .......*.. .......... .......... .*.....*.. ..........
OR504711.1 This study            .......... .......... .....*.... ..*.....*. .......*.. .......... .......... .*.....*.. ..........
OR504712.1 This study            .......... .......... .....*.... ..*.....*. .......*.. .......... .......... .*.....*.. ..........
OR504713.1 This study            .......... .......... .....*.... ..*.....*. .......*.. .......... .......... .*.....*.. ..........
OR504714.1 This study            .......... .......... .....*.... ..*.....*. .......*.. .......... .......... .*.....*.. ..........
OR504715.1 This study            .......... .......... .....*.... ..*.....*. .......*.. .......... .......... .*.....*.. ..........
OR504716.1 This study            .......... .......... .....*.... ..*.....*. .......*.. .......... .......... .*.....*.. ..........
```

```
                                         100        110        120        130        140        150        160        170        180
                                 ....|....| ....|....| ....|....| ....|....| ....|....| ....|....| ....|....| ....|....| ....|....|
NC013590.2 (C-27)-Reference      GGIDIRAGYR RYYSNHTTIS KKNXX*RDYK LV*GNPGL*I PYVSYXI*DF VILNGNLEYV LYGRLHIGWN L*QSICS*XD DELGLIMMAP
OL410296.1 Marial vaccine        .......... .......... .....*.... ..*.....*. .......*.. .......... .......... .*.....*.. ..........
KR296657.1 Virbac vaccine        .......... .......... .....*.... ..*.....*. .......*.. .......... .......... .*.....*.. ..........
KR381803.1 Intervet vaccine      .......... .......... .....*.... ..*.....*. .......*.. .......... .......... .*.....*.. ..........
MH070348.1(KANS-02)              .......... .......... .....*.... ..*.....*. .......*.. .......... .......... .*.....*.. ..........
OR504717.1 This study            .......... .......... .....*.... ..*.....*. .......*.. .......... .......... .*.....*.. ..........
OR504718.1 This study            .......... .......... .....*.... ..*.....*. .......*.. .......... .......... .*.....*.. ..........
OR504719.1 This study            .......... .......... .....*.... ..*.....*. .......*.. .......... .......... .*.....*.. ..........
OR504720.1 This study            .......... .......... .....*.... ..*.....*. .......*.. .......... .......... .*.....*.. ..........
OR504721.1 This study            .......... .......... .....*.... ..*.....*. .......*.. .......... .......... .*.....*.. ..........
OR504722.1 This study            .......... .......... .....*.... ..*.....*. .......*.. .......... .......... .*.....*.. ..........
OR504723.1 This study            .......... .......... .....*.... ..*.....*. .......*.. .......... .......... .*.....*.. ..........
OR504724.1 This study            .......... .......... .....*.... ..*.....*. .......*.. .......... .......... .*.....*.. ..........
OR504725.1 This study            .......... .......... .....*.... ..*.....*. .......*.. .......... .......... .*.....*.. ..........
OR504726.1 This study            .......... .......... .....*.... ..*.....*. .......*.. .......... .......... .*.....*.. ..........
OR504727.1 This study            .......... .......... .....*.... ..*.....*. .......*.. .......... .......... .*.....*.. ..........
```

```
                                                    100        110        120        130        140        150        160        170        180
                                  ....|....| ....|....| ....|....| ....|....| ....|....| ....|....| ....|....| ....|....| ....|....|
NC013590.2 (C-27)-Reference       GGIDIRAGYR RYYSNHTTIS KKNXX*RDYK LV*GNPGL*I PYVSYXI*DF VILNGNLEYV LYGRLHIGWN L*QSICS*XD DELGLIMMAP
OL410296.1 Marial vaccine         .......... .......... .....*.... ..*.....*. .......*.. .......... .......... .*.....*.. ..........
KR296657.1 Virbac vaccine         .......... .......... .....*.... ..*.....*. .......*.. .......... .......... .*.....*.. ..........
KR381803.1 Intervet vaccine       .......... .......... .....*.... ..*.....*. .......*.. .......... .......... .*.....*.. ..........
MH070348.1(KANS-02)               .......... .......... .....*.... ..*.....*. .......*.. .......... .......... .*.....*.. ..........
OR504728.1 This study             .......... .......... .....*.... ..*.....*. .......*.. .......... .......... .*.....*.. ..........
OR504729.1 This study             .......... .......... .....*.... ..*.....*. .......*.. .......... .......... .*.....*.. ..........
OR504730.1 This study             .......... .......... .....*.... ..*.....*. .......*.. .......... .......... .*.....*.. ..........
OR504731.1 This study             .......... .......... .....*.... ..*.....*. .......*.. .......... .......... .*.....*.. ..........
OR504732.1 This study             .......... .......... .....*.... ..*.....*. .......*.. .......... .......... .*.....*.. ..........
OR504733.1 This study             .......... .......... .....*.... ..*.....*. .......*.. .......... .......... .*.....*.. ..........
OR504734.1 This study             .......... .......... .....*.... ..*.....*. .......*.. .......... .......... .*.....*.. ..........
OR504735.1 This study             .......... .......... .....*.... ..*.....*. .......*.. .......... .......... .*.....*.. ..........
OR504736.1 This study             .......... .......... .....*.... ..*.....*. .......*.. .......... .......... .*.....*.. ..........
OR504737.1 This study             .......... .......... .....*.... ..*.....*. .......*.. .......... .......... .*.....*.. ..........
OR504738.1 This study             .......... .......... .....*.... ..*.....*. .......*.. .......... .......... .*.....*.. ..........
```

```
                                                    100        110        120        130        140        150        160        170        180
                                  ....|....| ....|....| ....|....| ....|....| ....|....| ....|....| ....|....| ....|....| ....|....|
NC013590.2 (C-27)-Reference       GGIDIRAGYR RYYSNHTTIS KKNXX*RDYK LV*GNPGL*I PYVSYXI*DF VILNGNLEYV LYGRLHIGWN L*QSICS*XD DELGLIMMAP
OL410296.1 Marial vaccine         .......... .......... .....*.... ..*.....*. .......*.. .......... .......... .*.....*.. ..........
KR296657.1 Virbac vaccine         .......... .......... .....*.... ..*.....*. .......*.. .......... .......... .*.....*.. ..........
KR381803.1 Intervet vaccine       .......... .......... .....*.... ..*.....*. .......*.. .......... .......... .*.....*.. ..........
MH070348.1(KANS-02)               .......... .......... .....*.... ..*.....*. .......*.. .......... .......... .*.....*.. ..........
OR504739.1 This study             .......... .......... .....*.... ..*.....*. .......*.. .......... .......... .*.....*.. ..........
OR504740.1 This study             .......... .......... .....*.... ..*.....*. .......*.. .......... .......... .*.....*.. ..........
OR504741.1 This study             .......... .......... .....*.... ..*.....*. .......*.. .......... .......... .*.....*.. ..........
OR504742.1 This study             .......... .......... .....*.... ..*.....*. .......*.. .......... .......... .*.....*.. ..........
OR504743.1 This study             .......... .......... .....*.... ..*.....*. .......*.. .......... .......... .*.....*.. ..........
OR504744.1 This study             .......... .......... .....*.... ..*.....*. .......*.. .......... .......... .*.....*.. ..........
OR504745.1 This study             .......... .......... .....*.... ..*.....*. .......*.. .......... .......... .*.....*.. ..........
OR504746.1 This study             .......... .......... .....*.... ..*.....*. .......*.. .......... .......... .*.....*.. ..........
OR504747.1 This study             .......... .......... .....*.... ..*.....*. .......*.. .......... .......... .*.....*.. ..........
OR504748.1 This study             .......... .......... .....*.... ..*.....*. .......*.. .......... .......... .*.....*.. ..........
```

```
                                                190        200        210        220        230        240        250        260        270
                                       ....|....| ....|....| ....|....| ....|....| ....|....| ....|....| ....|....| ....|....| ....|....|
NC013590.2 (C-27)-Reference            AQFNQGQYRR VITIDGSMFY TDFMVQLSPT PCWFAKPDRY EEILHEWCRN VKTIXXLMXS S*LPLLXXGT L*PTTSP*SR TLIXWYRTHG
OL410296.1 Marial vaccine              .......... .......... .......... .......... .......... .......... .*........ .*.....*.. ..........
KR296657.1 Virbac vaccine              .......... .......... .......... .......... .......... .......... .*........ .*.....*.. ..........
KR381803.1 Intervet vaccine            .......... .......... .......... .......... .......... .......... .*........ .*.....*.. ..........
MH070348.1(KANS-02)                    .......... .......... .......... .......... .......... .......... .*........ .*.....*.. ..........
OR504706.1 This study                  .......... .......... .......... .......... .......... .......... .*........ .*.....*.. ..........
OR504707.1 This study                  .......... .......... .......... .......... .......... .......... .*........ .*.....*.. ..........
OR504708.1 This study                  .......... .......... .......... .......... .......... .......... .*........ .*.....*.. ..........
OR504709.1 This study                  .......... .......... .......... .......... .......... .......... .*........ .*.....*.. ..........
OR504710.1 This study                  .......... .......... .......... .......... .......... .......... .*........ .*.....*.. ..........
OR504711.1 This study                  .......... .......... .......... .......... .......... .......... .*........ .*.....*.. ..........
OR504712.1 This study                  .......... .......... .......... .......... .......... .......... .*........ .*.....*.. ..........
OR504713.1 This study                  .......... .......... .......... .......... .......... .......... .*........ .*.....*.. ..........
OR504714.1 This study                  .......... .......... .......... .......... .......... .......... .*........ .*.....*.. ..........
OR504715.1 This study                  .......... .......... .......... .......... .......... .......... .*........ .*.....*.. ..........
OR504716.1 This study                  .......... .......... .......... .......... .......... .......... .*........ .*.....*.. ..........
```

```
                                                190        200        210        220        230        240        250        260        270
                                       ....|....| ....|....| ....|....| ....|....| ....|....| ....|....| ....|....| ....|....| ....|....|
NC013590.2 (C-27)-Reference            AQFNQGQYRR VITIDGSMFY TDFMVQLSPT PCWFAKPDRY EEILHEWCRN VKTIXXLMXS S*LPLLXXGT L*PTTSP*SR TLIXWYRTHG
OL410296.1 Marial vaccine              .......... .......... .......... .......... .......... .......... .*........ .*.....*.. ..........
KR296657.1 Virbac vaccine              .......... .......... .......... .......... .......... .......... .*........ .*.....*.. ..........
KR381803.1 Intervet vaccine            .......... .......... .......... .......... .......... .......... .*........ .*.....*.. ..........
MH070348.1(KANS-02)                    .......... .......... .......... .......... .......... .......... .*........ .*.....*.. ..........
OR504717.1 This study                  .......... .......... .......... .......... .......... .......... .*........ .*.....*.. ..........
OR504718.1 This study                  .......... .......... .......... .......... .......... .......... .*........ .*.....*.. ..........
OR504719.1 This study                  .......... .......... .......... .......... .......... .......... .*........ .*.....*.. ..........
OR504720.1 This study                  .......... .......... .......... .......... .......... .......... .*........ .*.....*.. ..........
OR504721.1 This study                  .......... .......... .......... .......... .......... .......... .*........ .*.....*.. ..........
OR504722.1 This study                  .......... .......... .......... .......... .......... .......... .*........ .*.....*.. ..........
OR504723.1 This study                  .......... .......... .......... .......... .......... .......... .*........ .*.....*.. ..........
OR504724.1 This study                  .......... .......... .......... .......... .......... .......... .*........ .*.....*.. ..........
OR504725.1 This study                  .......... .......... .......... .......... .......... .......... .*........ .*.....*.. ..........
OR504726.1 This study                  .......... .......... .......... .......... .......... .......... .*........ .*.....*.. ..........
OR504727.1 This study                  .......... .......... .......... .......... .......... .......... .*........ .*.....*.. ..........
```

```
                                    190        200        210        220        230        240        250        260        270
                             ....|....| ....|....| ....|....| ....|....| ....|....| ....|....| ....|....| ....|....| ....|....|
NC013590.2 (C-27)-Reference  AQFNQGQYRR VITIDGSMFY TDFMVQLSPT PCWFAKPDRY EEILHEWCRN VKTIXXLMXS S*LPLLXXGT L*PTTSP*SR TLIXWYRTHG
OL410296.1 Marial vaccine    .......... .......... .......... .......... .......... .......... .*........ .*.....*.. ..........
KR296657.1 Virbac vaccine    .......... .......... .......... .......... .......... .......... .*........ .*.....*.. ..........
KR381803.1 Intervet vaccine  .......... .......... .......... .......... .......... .......... .*........ .*.....*.. ..........
MH070348.1(KANS-02)          .......... .......... .......... .......... .......... .......... .*........ .*.....*.. ..........
OR504728.1 This study        .......... .......... .......... .......... .......... .......... .*........ .*.....*.. ..........
OR504729.1 This study        .......... .......... .......... .......... .......... .......... .*........ .*.....*.. ..........
OR504730.1 This study        .......... .......... .......... .......... .......... .......... .*........ .*.....*.. ..........
OR504731.1 This study        .......... .......... .......... .......... .......... .......... .*........ .*.....*.. ..........
OR504732.1 This study        .......... .......... .......... .......... .......... .......... .*........ .*.....*.. ..........
OR504733.1 This study        .......... .......... .......... .......... .......... .......... .*........ .*.....*.. ..........
OR504734.1 This study        .......... .......... .......... .......... .......... .......... .*........ .*.....*.. ..........
OR504735.1 This study        .......... .......... .......... .......... .......... .......... .*........ .*.....*.. ..........
OR504736.1 This study        .......... .......... .......... .......... .......... .......... .*........ .*.....*.. ..........
OR504737.1 This study        .......... .......... .......... .......... .......... .......... .*........ .*.....*.. ..........
OR504738.1 This study        .......... .......... .......... .......... .......... .......... .*........ .*.....*.. ..........
```

```
                                    190        200        210        220        230        240        250        260        270
                             ....|....| ....|....| ....|....| ....|....| ....|....| ....|....| ....|....| ....|....| ....|....|
NC013590.2 (C-27)-Reference  AQFNQGQYRR VITIDGSMFY TDFMVQLSPT PCWFAKPDRY EEILHEWCRN VKTIXXLMXS S*LPLLXXGT L*PTTSP*SR TLIXWYRTHG
OL410296.1 Marial vaccine    .......... .......... .......... .......... .......... .......... .*........ .*.....*.. ..........
KR296657.1 Virbac vaccine    .......... .......... .......... .......... .......... .......... .*........ .*.....*.. ..........
KR381803.1 Intervet vaccine  .......... .......... .......... .......... .......... .......... .*........ .*.....*.. ..........
MH070348.1(KANS-02)          .......... .......... .......... .......... .......... .......... .*........ .*.....*.. ..........
OR504739.1 This study        .......... .......... .......... .......... .......... .......... .*........ .*.....*.. ..........
OR504740.1 This study        .......... .......... .......... .......... .......... .......... .*........ .*.....*.. ..........
OR504741.1 This study        .......... .......... .......... .......... .......... .......... .*........ .*.....*.. ..........
OR504742.1 This study        .......... .......... .......... .......... .......... .......... .*........ .*.....*.. ..........
OR504743.1 This study        .......... .......... .......... .......... .......... .......... .*........ .*.....*.. ..........
OR504744.1 This study        .......... .......... .......... .......... .......... .......... .*........ .*.....*.. ..........
OR504745.1 This study        .......... .......... .......... .......... .......... .......... .*........ .*.....*.. ..........
OR504746.1 This study        .......... .......... .......... .......... .......... .......... .*........ .*.....*.. ..........
OR504747.1 This study        .......... .......... .......... .......... .......... .......... .*........ .*.....*.. ..........
OR504748.1 This study        .......... .......... .......... .......... .......... .......... .*........ .*.....*.. ..........
```

```
                                         280        290        300        310        320        330        340        350        360
                                 ....|....| ....|....| ....|....| ....|....| ....|....| ....|....| ....|....| ....|....| ....|....|
NC013590.2 (C-27)-Reference      REPPVRFQEA IRYDRPAIPS GSEDSKRSND SRGESSGPNW IDIEXITXPK NNVPIIISDD DVPTAPPKGX XNNQXXVVIP AIVLXLXXII
OL410296.1 Marial vaccine        .......... .......... .......... .......... .......... .......... .......... .......... ..........
KR296657.1 Virbac vaccine        .......... .......... .......... .......... .......... .......... .......... .......... ..........
KR381803.1 Intervet vaccine      .......... .......... .......... .......... .......... .......... .......... .......... ..........
MH070348.1(KANS-02)              .......... .......... .......... .......... .......... .......... .......... .......... ..........
OR504706.1 This study            .......... .......... .......... .......... .......... .......... .......... .......... ..........
OR504707.1 This study            .......... .......... .......... .......... .......... .......... .......... .......... ..........
OR504708.1 This study            .......... .......... .......... .......... .......... .......... .......... .......... ..........
OR504709.1 This study            .......... .......... .......... .......... .......... .......... .......... .......... ..........
OR504710.1 This study            .......... .......... .......... .......... .......... .......... .......... .......... ..........
OR504711.1 This study            .......... .......... .......... .......... .......... .......... .......... .......... ..........
OR504712.1 This study            .......... .......... .......... .......... .......... .......... .......... .......... ..........
OR504713.1 This study            .......... .......... .......... .......... .......... .......... .......... .......... ..........
OR504714.1 This study            .......... .......... .......... .......... .......... .......... .......... .......... ..........
OR504715.1 This study            .......... .......... .......... .......... .......... .......... .......... .......... ..........
OR504716.1 This study            .......... .......... .......... .......... .......... .......... .......... .......... ..........
```

```
                                         280        290        300        310        320        330        340        350        360
                                 ....|....| ....|....| ....|....| ....|....| ....|....| ....|....| ....|....| ....|....| ....|....|
NC013590.2 (C-27)-Reference      REPPVRFQEA IRYDRPAIPS GSEDSKRSND SRGESSGPNW IDIEXITXPK NNVPIIISDD DVPTAPPKGX XNNQXXVVIP AIVLXLXXII
OL410296.1 Marial vaccine        .......... .......... .......... .......... .......... .......... .......... .......... ..........
KR296657.1 Virbac vaccine        .......... .......... .......... .......... .......... .......... .......... .......... ..........
KR381803.1 Intervet vaccine      .......... .......... .......... .......... .......... .......... .......... .......... ..........
MH070348.1(KANS-02)              .......... .......... .......... .......... .......... .......... .......... .......... ..........
OR504717.1 This study            .......... .......... .......... .......... .......... .......... .......... .......... ..........
OR504718.1 This study            .......... .......... .......... .......... .......... .......... .......... .......... ..........
OR504719.1 This study            .......... .......... .......... .......... .......... .......... .......... .......... ..........
OR504720.1 This study            .......... .......... .......... .......... .......... .......... .......... .......... ..........
OR504721.1 This study            .......... .......... .......... .......... .......... .......... .......... .......... ..........
OR504722.1 This study            .......... .......... .......... .......... .......... .......... .......... .......... ..........
OR504723.1 This study            .......... .......... .......... .......... .......... .......... .......... .......... ..........
OR504724.1 This study            .......... .......... .......... .......... .......... .......... .......... .......... ..........
OR504725.1 This study            .......... .......... .......... .......... .......... .......... .......... .......... ..........
OR504726.1 This study            .......... .......... .......... .......... .......... .......... .......... .......... ..........
OR504727.1 This study            .......... .......... .......... .......... .......... .......... .......... .......... ..........
```

```
                                     280        290        300        310        320        330        340        350        360
                                 ....|....| ....|....| ....|....| ....|....| ....|....| ....|....| ....|....| ....|....| ....|....|
NC013590.2 (C-27)-Reference      REPPVRFQEA IRYDRPAIPS GSEDSKRSND SRGESSGPNW IDIEXITXPK NNVPIIISDD DVPTAPPKGX XNNQXXVVIP AIVLXLXXII
OL410296.1 Marial vaccine        .......... .......... .......... .......... .......... .......... .......... .......... ..........
KR296657.1 Virbac vaccine        .......... .......... .......... .......... .......... .......... .......... .......... ..........
KR381803.1 Intervet vaccine      .......... .......... .......... .......... .......... .......... .......... .......... ..........
MH070348.1(KANS-02)              .......... .......... .......... .......... .......... .......... .......... .......... ..........
OR504728.1 This study            .......... .......... .......... .......... .......... .......... .......... .......... ..........
OR504729.1 This study            .......... .......... .......... .......... .......... .......... .......... .......... ..........
OR504730.1 This study            .......... .......... .......... .......... .......... .......... .......... .......... ..........
OR504731.1 This study            .......... .......... .......... .......... .......... .......... .......... .......... ..........
OR504732.1 This study            .......... .......... .......... .......... .......... .......... .......... .......... ..........
OR504733.1 This study            .......... .......... .......... .......... .......... .......... .......... .......... ..........
OR504734.1 This study            .......... .......... .......... .......... .......... .......... .......... .......... ..........
OR504735.1 This study            .......... .......... .......... .......... .......... .......... .......... .......... ..........
OR504736.1 This study            .......... .......... .......... .......... .......... .......... .......... .......... ..........
OR504737.1 This study            .......... .......... .......... .......... .......... .......... .......... .......... ..........
OR504738.1 This study            .......... .......... .......... .......... .......... .......... .......... .......... ..........
```

```
                                     280        290        300        310        320        330        340        350        360
                                 ....|....| ....|....| ....|....| ....|....| ....|....| ....|....| ....|....| ....|....| ....|....|
NC013590.2 (C-27)-Reference      REPPVRFQEA IRYDRPAIPS GSEDSKRSND SRGESSGPNW IDIEXITXPK NNVPIIISDD DVPTAPPKGX XNNQXXVVIP AIVLXLXXII
OL410296.1 Marial vaccine        .......... .......... .......... .......... .......... .......... .......... .......... ..........
KR296657.1 Virbac vaccine        .......... .......... .......... .......... .......... .......... .......... .......... ..........
KR381803.1 Intervet vaccine      .......... .......... .......... .......... .......... .......... .......... .......... ..........
MH070348.1(KANS-02)              .......... .......... .......... .......... .......... .......... .......... .......... ..........
OR504739.1 This study            .......... .......... .......... .......... .......... .......... .......... .......... ..........
OR504740.1 This study            .......... .......... .......... .......... .......... .......... .......... .......... ..........
OR504741.1 This study            .......... .......... .......... .......... .......... .......... .......... .......... ..........
OR504742.1 This study            .......... .......... .......... .......... .......... .......... .......... .......... ..........
OR504743.1 This study            .......... .......... .......... .......... .......... .......... .......... .......... ..........
OR504744.1 This study            .......... .......... .......... .......... .......... .......... .......... .......... ..........
OR504745.1 This study            .......... .......... .......... .......... .......... .......... .......... .......... ..........
OR504746.1 This study            .......... .......... .......... .......... .......... .......... .......... .......... ..........
OR504747.1 This study            .......... .......... .......... .......... .......... .......... .......... .......... ..........
OR504748.1 This study            .......... .......... .......... .......... .......... .......... .......... .......... ..........
```
