# Supplementary material for: First report of molecular epidemiology and phylogenetic characteristics of feline herpesvirus (FHV-1) from naturally infected cats in Kunshan, China
Source: Virol J. 2024 May 22;21:115. doi: 10.1186/s12985-024-02391-1 (PMC11112849; doi:10.1186/s12985-024-02391-1)
Supplement: Supplementary file 3 — Supplementary Material 3 [file 12985_2024_2391_MOESM3_ESM.pdf]

```
                                         10         20         30         40         50         60         70         80         90
                                ....|....| ....|....| ....|....| ....|....| ....|....| ....|....| ....|....| ....|....| ....|....|
NC013590.2 (C-27)-Reference     DSHVSILXGX EXMEPM*NXT SXILQTDFNK SRDTIQ*TWI RDYNWGHQFL XNFLETPHVT VAWXXWTPKS GXGMYLSQME GNR*NATR*X
OL410296.1 Marial vaccine       .......... ......*... .......... ......*... .......... .......... .......... .......... ...*....*.
KR296657.1 Virbac vaccine       .......... ......*... .......... ......*... .......... .......... .......... .......... ...*....*.
KR381803.1 Intervet vaccine     .......... ......*... .......... ......*... .......... .......... .......... .......... ...*....*.
MH070348.1 (KANS-02)            .......... ......*... .......... ......*... .......... .......... .......... .......... ...*....*.
OR504663.1 This study           .......... ......*... .......... ......*... .......... .......... .......... .......... ...*....*.
OR504664.1 This study           .......... ......*... .......... ......*... .......... .......... .......... .......... ...*....*.
OR504665.1 This study           .......... ......*..I .......... ......*... .......... .......... .......... .......... ...*....*.
OR504666.1 This study           .......... ......*... .......... ......*... .......... .......... .......... .......... ...*....*.
OR504667.1 This study           .......... ......*... .......... ......*... .......... .......... .......... .......... ...*....*.
OR504668.1 This study           .......... ......*... .......... ......*... .......... .......... .......... .......... ...*....*.
OR504669.1 This study           .......... ......*... .......... ......*... .......... .......... .......... .......... ...*....*.
OR504670.1 This study           .......... ......*... .......... ......*... .......... .......... .......... .......... ...*....*.
OR504671.1 This study           .......... ......*... .......... ......*... .......... .......... .......... .......... ...*....*.
OR504672.1 This study           .......... ......*... .......... ......*... .......... .......... .......... .......... ...*....*.
OR504673.1 This study           .......... ......*... .......... ......*... .......... .......... .......... .......... ...*....*.
```

```
                                         10         20         30         40         50         60         70         80         90
                                ....|....| ....|....| ....|....| ....|....| ....|....| ....|....| ....|....| ....|....| ....|....|
NC013590.2 (C-27)-Reference     DSHVSILXGX EXMEPM*NXT SXILQTDFNK SRDTIQ*TWI RDYNWGHQFL XNFLETPHVT VAWXXWTPKS GXGMYLSQME GNR*NATR*X
OL410296.1 Marial vaccine       .......... ......*... .......... ......*... .......... .......... .......... .......... ...*....*.
KR296657.1 Virbac vaccine       .......... ......*... .......... ......*... .......... .......... .......... .......... ...*....*.
KR381803.1 Intervet vaccine     .......... ......*... .......... ......*... .......... .......... .......... .......... ...*....*.
MH070348.1 (KANS-02)            .......... ......*... .......... ......*... .......... .......... .......... .......... ...*....*.
OR504673.1 This study           .......... ......*... .......... ......*... .......... .......... .......... .......... ...*....*.
OR504674.1 This study           .......... ......*... .......... ......*... .......... .......... .......... .......... ...*....*.
OR504675.1 This study           .......... ......*... .......... ......*... .......... .......... .......... .......... ...*....*.
OR504676.1 This study           .......... ......*... .......... ......*... .......... .......... .......... .......... ...*....*.
OR504677.1 This study           .......... ......*... ..F....... ......*... .......... .......... .......... .......... ...*....*.
OR504678.1 This study           .......... ......*... .......... ......*... .......... .......... .......... .......... ...*....*.
OR504679.1 This study           .......... ......*... .......... ......*... .......... .......... .......... .......... ...*....*.
OR504680.1 This study           .......... ......*... .......... ......*... .......... .......... .......... .......... ...*....*.
OR504681.1 This study           .......... ......*... .......... ......*... .......... .......... .......... .......... ...*....*.
OR504682.1 This study           .......... ......*... .......... ......*... .......... .......... .......... .......... ...*....*.
OR504683.1 This study           .......... ......*... ..F....... ......*... .......... .......... .......... .......... ...*....*.
```

```
                                       10         20         30         40         50         60         70         80         90
                                ....|....| ....|....| ....|....| ....|....| ....|....| ....|....| ....|....| ....|....| ....|....|
NC013590.2 (C-27)-Reference     DSHVSILXGX EXMEPM*NXT SXILQTDFNK SRDTIQ*TWI RDYNWGHQFL XNFLETPHVT VAWXXWTPKS GXGMYLSQME GNR*NATR*X
OL410296.1 Marial vaccine       .......... ......*... .......... ......*... .......... .......... .......... .......... ...*....*.
KR296657.1 Virbac vaccine       .......... ......*... .......... ......*... .......... .......... .......... .......... ...*....*.
KR381803.1 Intervet vaccine     .......... ......*... .......... ......*... .......... .......... .......... .......... ...*....*.
MH070348.1 (KANS-02)            .......... ......*... .......... ......*... .......... .......... .......... .......... ...*....*.
OR504684.1 This study           .......... ......*... .......... ......*... .......... .......... .......... .......... ...*....*.
OR504685.1 This study           .......... ......*... .......... ......*... .......... .......... .......... .......... ...*....*.
OR504686.1 This study           .......... ......*... .......... ......*... .......... .......... .......... .......... ...*....*.
OR504687.1 This study           .......... ......*... .......... ......*... .......... .......... .......... .......... ...*....*.
OR504688.1 This study           .......... ......*... .......... ......*... .......... .......... .......... .......... ...*....*.
OR504689.1 This study           .......... ......*... .......... ......*... .......... .......... .......... .......... ...*....*.
OR504690.1 This study           .......... ......*... .......... ......*... .......... .......... .......... .......... ...*....*.
OR504691.1 This study           .......... ......*... .......... ......*... .......... .......... .......... .......... ...*....*.
OR504692.1 This study           .......... ......*... .......... ......*... .......... .......... .......... .......... ...*....*.
OR504693.1 This study           .......... ......*... .......... ......*... .......... .......... .......... .......... ...*....*.
OR504694.1 This study           .......... ......*... .......... ......*... .......... .......... .......... .......... ...*....*.
```

```
                                       10         20         30         40         50         60         70         80         90
                                ....|....| ....|....| ....|....| ....|....| ....|....| ....|....| ....|....| ....|....| ....|....|
NC013590.2 (C-27)-Reference     DSHVSILXGX EXMEPM*NXT SXILQTDFNK SRDTIQ*TWI RDYNWGHQFL XNFLETPHVT VAWXXWTPKS GXGMYLSQME GNR*NATR*X
OL410296.1 Marial vaccine       .......... ......*... .......... ......*... .......... .......... .......... .......... ...*....*.
KR296657.1 Virbac vaccine       .......... ......*... .......... ......*... .......... .......... .......... .......... ...*....*.
KR381803.1 Intervet vaccine     .......... ......*... .......... ......*... .......... .......... .......... .......... ...*....*.
MH070348.1 (KANS-02)            .......... ......*... .......... ......*... .......... .......... .......... .......... ...*....*.
OR504695.1 This study           .......... ......*... .......... ......*... .......... .......... .......... .......... ...*....*.
OR504696.1 This study           .......... ......*... .......... ......*... .......... .......... .......... .......... ...*....*.
OR504697.1 This study           .......... ......*... .......... ......*... .......... .......... .......... .......... ...*....*.
OR504698.1 This study           .......... ......*... .F........ ......*... .......... .......... .......... .......... ...*....*.
OR504699.1 This study           .......... ......*... .......... ......*... .......... .......... .......... .......... ...*....*.
OR504700.1 This study           .......... ......*... .......... ......*... .......... .......... .......... .......... ...*....*.
OR504701.1 This study           .......... ......*... .......... ......*... .......... .......... .......... .......... ...*....*.
OR504702.1 This study           .......... ......*... .......... ......*... .......... .......... .......... .......... ...*....*.
OR504703.1 This study           .......... ......*... .......... ......*... .......... .......... .......... .......... ...*....*.
OR504704.1 This study           .......... ......*... .......... ......*... .......... .......... .......... .......... ...*....*.
OR504705.1 This study           .......... ......*... .......... ......*... .......... .......... .......... .......... ...*....*.
```

```
                                        100        110        120        130        140        150        160        170        180
                                ....|....| ....|....| ....|....| ....|....| ....|....| ....|....| ....|....| ....|....| ....|....|
NC013590.2 (C-27)-Reference     XSGLXIDLQP RXISATFISN XFTIXKSIVS VWGTVPPRRQ PXAIDRIYKS KYSKXSYSDW XTLETYLARG GXF**LSXPM IXXQXELAKL
OL410296.1 Marial vaccine       .......... .......... .......... .......... .......... .......... .......... ...**..... ..........
KR296657.1 Virbac vaccine       .......... .......... .......... .......... .......... .......... .......... ...**..... ..........
KR381803.1 Intervet vaccine     .......... .......... .......... .......... .......... .......... .......... ...**..... ..........
MH070348.1 (KANS-02)            .......... .......... .......... .......... .......... .......... .......... ...**..... ..........
OR504663.1 This study           .......... .......... .......... .......... .......... .......... .......... ...**..... ..........
OR504664.1 This study           .......... .......... .......... .......... .......... .......... .......... ...**..... ..........
OR504665.1 This study           .......... .......... .......... .......... .......... .......... .......... ...**..... ..........
OR504666.1 This study           .......... .......... .......... .......... .......... .......... .......... ...**..... ..........
OR504667.1 This study           .......... .......... .......... .......... .......... .......... .......... ...**..... ..........
OR504668.1 This study           .......... .......... .......... .......... .......... .......... .......... ...**..... ..........
OR504669.1 This study           .......... .......... .......... .......... .......... .......... .......... ...**..... ..........
OR504670.1 This study           .......... .......... .......... .......... .......... .......... .......... ...**..... ..........
OR504671.1 This study           .......... .......... .......... .......... .......... .......... .......... ...**..... ..........
OR504672.1 This study           .......... .......... .......... .......... .......... .......... .......... ...**..... ..........
OR504673.1 This study           .......... .......... .......... .......... .......... .......... .......... ...**..... ..........
```

```
                                        100        110        120        130        140        150        160        170        180
                                ....|....| ....|....| ....|....| ....|....| ....|....| ....|....| ....|....| ....|....| ....|....|
NC013590.2 (C-27)-Reference     XSGLXIDLQP RXISATFISN XFTIXKSIVS VWGTVPPRRQ PXAIDRIYKS KYSKXSYSDW XTLETYLARG GXF**LSXPM IXXQXELAKL
OL410296.1 Marial vaccine       .......... .......... .......... .......... .......... .......... .......... ...**..... ..........
KR296657.1 Virbac vaccine       .......... .......... .......... .......... .......... .......... .......... ...**..... ..........
KR381803.1 Intervet vaccine     .......... .......... .......... .......... .......... .......... .......... ...**..... ..........
MH070348.1 (KANS-02)            .......... .......... .......... .......... .......... .......... .......... ...**..... ..........
OR504673.1 This study           .......... .......... .......... .......... .......... .......... .......... ...**..... ..........
OR504674.1 This study           .......... .......... .......... .......... .......... .......... .......... ...**..... ..........
OR504675.1 This study           .......... .......... .......... .......... .......... .......... .......... ...**..... ..........
OR504676.1 This study           .......... .......... .......... .......... .......... .......... .......... ...**..... ..........
OR504677.1 This study           .......... .......... .......... .......... .......... .......... .......... ...**..... ..........
OR504678.1 This study           .......... .......... .......... .......... .......... .......... .......... ...**..... ..........
OR504679.1 This study           .......... .......... .......... .......... .......... .......... .......... ...**..... ..........
OR504680.1 This study           .......... .......... .......... .......... .......... .......... .......... ...**..... ..........
OR504681.1 This study           .......... .......... .......... .......... .......... .......... .......... ...**..... ..........
OR504682.1 This study           .......... .......... .......... .......... .......... .......... .......... ...**..... ..........
OR504683.1 This study           .......... .......... .......... .......... .......... .......... .......... ...**..... ..........
```

```
                                      100        110        120        130        140        150        160        170        180
                               ....|....| ....|....| ....|....| ....|....| ....|....| ....|....| ....|....| ....|....| ....|....|
NC013590.2 (C-27)-Reference    XSGLXIDLQP RXISATFISN XFTIXKSIVS VWGTVPPRRQ PXAIDRIYKS KYSKXSYSDW XTLETYLARG GXF**LSXPM IXXQXELAKL
OL410296.1 Marial vaccine      .......... .......... .......... .......... .......... .......... .......... ...**..... ..........
KR296657.1 Virbac vaccine      .......... .......... .......... .......... .......... .......... .......... ...**..... ..........
KR381803.1 Intervet vaccine    .......... .......... .......... .......... .......... .......... .......... ...**..... ..........
MH070348.1 (KANS-02)           .......... .......... .......... .......... .......... .......... .......... ...**..... ..........
OR504684.1 This study          .......... .......... .......... .......... .......... .......... .......... ...**..... ..........
OR504685.1 This study          .......... .......... .......... .......... .......... .......... .......... ...**..... ..........
OR504686.1 This study          .......... .......... .......... .......... .......... .......... .......... ...**..... ..........
OR504687.1 This study          .......... .......... .......... .......... .......... .......... .......... ...**..... ..........
OR504688.1 This study          .......... .......... .......... .......... .......... .......... .......... ...**..... ..........
OR504689.1 This study          .......... .......... .......... .......... .......... .......... .......... ...**..... ..........
OR504690.1 This study          .......... .......... .......... .......... .......... .......... .......... ...**..... ..........
OR504691.1 This study          .......... .......... .......... .......... .......... .......... .......... ...**..... ..........
OR504692.1 This study          .......... .......... .......... .......... .......... .......... .......... ...**..... ..........
OR504693.1 This study          .......... .......... .......... .......... .......... .......... .......... ...**..... ..........
OR504694.1 This study          .......... .......... .......... .......... .......... .......... .......... ...**..... ..........
```

```
                                      100        110        120        130        140        150        160        170        180
                               ....|....| ....|....| ....|....| ....|....| ....|....| ....|....| ....|....| ....|....| ....|....|
NC013590.2 (C-27)-Reference    XSGLXIDLQP RXISATFISN XFTIXKSIVS VWGTVPPRRQ PXAIDRIYKS KYSKXSYSDW XTLETYLARG GXF**LSXPM IXXQXELAKL
OL410296.1 Marial vaccine      .......... .......... .......... .......... .......... .......... .......... ...**..... ..........
KR296657.1 Virbac vaccine      .......... .......... .......... .......... .......... .......... .......... ...**..... ..........
KR381803.1 Intervet vaccine    .......... .......... .......... .......... .......... .......... .......... ...**..... ..........
MH070348.1 (KANS-02)           .......... .......... .......... .......... .......... .......... .......... ...**..... ..........
OR504695.1 This study          .......... .......... .......... .......... .......... .......... .......... ...**..... ..........
OR504696.1 This study          .......... .......... .......... .......... .......... .......... .......... ...**..... ..........
OR504697.1 This study          .......... .......... .......... .......... .......... .......... .......... ...**..... ..........
OR504698.1 This study          .......... .......... .......... .......... .......... .......... .......... ...**..... ..........
OR504699.1 This study          .......... .......... .......... .......... .......... .......... .......... ...**..... ..........
OR504700.1 This study          .......... .......... .......... .......... .......... .......... .......... ...**..... ..........
OR504701.1 This study          .......... .......... .......... .......... .......... .......... .......... ...**..... ..........
OR504702.1 This study          .......... .......... .......... .......... .......... .......... .......... ...**..... ..........
OR504703.1 This study          .......... .......... .......... .......... .......... .......... .......... ...**..... ..........
OR504704.1 This study          .......... .......... .......... .......... .......... .......... .......... ...**..... ..........
OR504705.1 This study          .......... .......... .......... .......... .......... .......... .......... ...**..... ..........
```

```
                                          190       200       210       220       230       240       250       260       270
                               ....|....| ....|....| ....|....| ....|....| ....|....| ....|....| ....|....| ....|....| ....|....|
NC013590.2 (C-27)-Reference    YXQ*ISTFQS HX*ISVHSSX PSGETXXQRT RRSVPSNQHH XGXAXXQXQ* RGXIETVNXX CIXLLXDHLI XVEFAMXTIC LXTXXTSPCK
OL410296.1 Marial vaccine      ...*...... ..*....... .......... .......... .........* .......... .......... .......... ..........
KR296657.1 Virbac vaccine      ...*...... ..*....... .......... .......... .........* .......... .......... .......... ..........
KR381803.1 Intervet vaccine    ...*...... ..*....... .......... .......... .........* .......... .......... .......... ..........
MH070348.1 (KANS-02)           ...*...... ..*....... .......... .......... .........* .......... .......... .......... ..........
OR504663.1 This study          ...*...... ..*....... .......... .......... .........* .......... .......... .......... ..........
OR504664.1 This study          ...*...... ..*....... .......... .......... .........* .......... .......... .......... ..........
OR504665.1 This study          ...*...... ..*....... .......... .......... .........* .......... .......... .......... ..........
OR504666.1 This study          ...*...... ..*....... .......... .......... .........* .......... .......... .......... ..........
OR504667.1 This study          ...*...... ..*....... .......... .......... .........* .......... .......... .......... ..........
OR504668.1 This study          ...*...... ..*....... .......... .......... .........* .......... .......... .......... ..........
OR504669.1 This study          ...*...... ..*....... .......... .......... .........* .......... .......... .......... ..........
OR504670.1 This study          ...*...... ..*....... .......... .......... .........* .......... .......... .......... ..........
OR504671.1 This study          ...*...... ..*....... .......... .......... .........* .......... .......... .......... ..........
OR504672.1 This study          ...*...... ..*....... .......... .......... .........* .......... .......... .......... ..........
OR504673.1 This study          ...*...... ..*....... .......... .......... .........* .......... .......... .......... ..........
```

```
                                          190       200       210       220       230       240       250       260       270
                               ....|....| ....|....| ....|....| ....|....| ....|....| ....|....| ....|....| ....|....| ....|....|
NC013590.2 (C-27)-Reference    YXQ*ISTFQS HX*ISVHSSX PSGETXXQRT RRSVPSNQHH XGXAXXQXQ* RGXIETVNXX CIXLLXDHLI XVEFAMXTIC LXTXXTSPCK
OL410296.1 Marial vaccine      ...*...... ..*....... .......... .......... .........* .......... .......... .......... ..........
KR296657.1 Virbac vaccine      ...*...... ..*....... .......... .......... .........* .......... .......... .......... ..........
KR381803.1 Intervet vaccine    ...*...... ..*....... .......... .......... .........* .......... .......... .......... ..........
MH070348.1 (KANS-02)           ...*...... ..*....... .......... .......... .........* .......... .......... .......... ..........
OR504673.1 This study          ...*...... ..*....... .......... .......... .........* .......... .......... .......... ..........
OR504674.1 This study          ...*...... ..*....... .......... .......... .........* .......... .......... .......... ..........
OR504675.1 This study          ...*...... ..*....... .......... .......... .........* .......... .......... .......... ..........
OR504676.1 This study          ...*...... ..*....... .......... .......... .........* .......... .......... .......... ..........
OR504677.1 This study          ...*...... ..*....... .......... .......... .........* .......... .......... .......... ..........
OR504678.1 This study          ...*...... ..*....... .......... .......... .........* .......... .......... .......... ..........
OR504679.1 This study          ...*...... ..*....... .......... .......... .........* .......... .......... .......... ..........
OR504680.1 This study          ...*...... ..*....... .......... .......... .........* .......... .......... .......... ..........
OR504681.1 This study          ...*...... ..*....... .......... .......... .........* .......... .......... .......... ..........
OR504682.1 This study          ...*...... ..*....... .......... .......... .........* .......... .......... .......... ..........
OR504683.1 This study          ...*...... ..*....... .......... .......... .........* .......... .......... .......... ..........
```

```
                                         190       200       210       220       230       240       250       260       270
                                  ....|....| ....|....| ....|....| ....|....| ....|....| ....|....| ....|....| ....|....| ....|....|
NC013590.2 (C-27)-Reference       YXQ*ISTFQS HX*ISVHSSX PSGETXXQRT RRSVPSNQHH XGXAXXQXQ* RGXIETVNXX CIXLLXDHLI XVEFAMXTIC LXTXXTSPCK
OL410296.1 Marial vaccine         ...*...... ..*....... .......... .......... .........* .......... .......... .......... ..........
KR296657.1 Virbac vaccine         ...*...... ..*....... .......... .......... .........* .......... .......... .......... ..........
KR381803.1 Intervet vaccine       ...*...... ..*....... .......... .......... .........* .......... .......... .......... ..........
MH070348.1 (KANS-02)              ...*...... ..*....... .......... .......... .........* .......... .......... .......... ..........
OR504684.1 This study             ...*...... ..*....... .......... .......... .........* .......... .......... .......... ..........
OR504685.1 This study             ...*...... ..*....... .......... .......... .........* .......... .......... .......... ..........
OR504686.1 This study             ...*...... ..*....... .......... .......... .........* .......... .......... .......... ..........
OR504687.1 This study             ...*...... ..*....... .......... .......... .........* .......... .......... .......... ..........
OR504688.1 This study             ...*...... ..*....... .......... .......... .........* .......... .......... .......... ..........
OR504689.1 This study             ...*...... ..*....... .......... .......... .........* .......... .......... .......... ..........
OR504690.1 This study             ...*...... ..*....... .......... .......... .........* .......... .......... .......... ..........
OR504691.1 This study             ...*...... ..*....... .......... .......... .........* .......... .......... .......... ..........
OR504692.1 This study             ...*...... ..*....... .......... .......... .........* .......... .......... .......... ..........
OR504693.1 This study             ...*...... ..*....... .......... .......... .........* .......... .......... .......... ..........
OR504694.1 This study             ...*...... ..*....... .......... .......... .........* .......... .......... .......... ..........
```

```
                                         190       200       210       220       230       240       250       260       270
                                  ....|....| ....|....| ....|....| ....|....| ....|....| ....|....| ....|....| ....|....| ....|....|
NC013590.2 (C-27)-Reference       YXQ*ISTFQS HX*ISVHSSX PSGETXXQRT RRSVPSNQHH XGXAXXQXQ* RGXIETVNXX CIXLLXDHLI XVEFAMXTIC LXTXXTSPCK
OL410296.1 Marial vaccine         ...*...... ..*....... .......... .......... .........* .......... .......... .......... ..........
KR296657.1 Virbac vaccine         ...*...... ..*....... .......... .......... .........* .......... .......... .......... ..........
KR381803.1 Intervet vaccine       ...*...... ..*....... .......... .......... .........* .......... .......... .......... ..........
MH070348.1 (KANS-02)              ...*...... ..*....... .......... .......... .........* .......... .......... .......... ..........
OR504695.1 This study             ...*...... ..*....... .......... .......... .........* .......... .......... .......... ..........
OR504696.1 This study             ...*...... ..*....... .......... .......... .........* .......... .......... .......... ..........
OR504697.1 This study             ...*...... ..*....... .......... .......... .........* .......... .......... .......... ..........
OR504698.1 This study             ...*...... ..*....... .......... .......... .........* .......... .......... .......... ..........
OR504699.1 This study             ...*...... ..*....... .......... .......... .........* .......... .......... .......... ..........
OR504700.1 This study             ...*...... ..*....... .......... .......... .........* .......... .......... .......... ..........
OR504701.1 This study             ...*...... ..*....... .......... .......... .........* .......... .......... .......... ..........
OR504702.1 This study             ...*...... ..*....... .......... .......... .........* .......... .......... .......... ..........
OR504703.1 This study             ...*...... ..*....... .......... .......... .........* .......... .......... .......... ..........
OR504704.1 This study             ...*...... ..*....... .......... .......... .........* .......... .......... .......... ..........
OR504705.1 This study             ...*...... ..*....... .......... .......... .........* .......... .......... .......... ..........
```

```
                                    280       290       300       310       320       330       340       350
                              ....|....| ....|....| ....|....| ....|....| ....|....| ....|....| ....|....| ....|....| ...
NC013590.2 (C-27)-Reference   *NVESDSHCL VYTSEXXANM CCGQRP*NSI PVXXGLDGPR TSCIXXRAYL EMPSXXNTMC *XFX*RTCLY XKILCG*RVH QRX
OL410296.1 Marial vaccine     *......... .......... ......*... .......... .......... .......... *...*..... ......*... ...
KR296657.1 Virbac vaccine     *......... .......... ......*... .......... .......... .......... *...*..... ......*... ...
KR381803.1 Intervet vaccine   *......... .......... ......*... .......... .......... .......... *...*..... ......*... ...
MH070348.1 (KANS-02)          *......... .......... ......*... .......... .......... .......... *...*..... ......*... ...
OR504663.1 This study         *......... .......... ......*... .......... .......... .......... *...*..... ......*... ...
OR504664.1 This study         *......... .......... ......*... .......... .......... .......... *...*P.... ......*... ...
OR504665.1 This study         *......... .......... ......*... .......... .......... .......... *...*P.F.. ......*... ...
OR504666.1 This study         *......... .......... ......*... .......... .......... .......... *...*..... ......*... ...
OR504667.1 This study         *......... .......... ......*... .......... .......... .......... *...*..... ......*... ...
OR504668.1 This study         *......... .......... ......*... .......... .......... .......... *...*..... ......*... ...
OR504669.1 This study         *......... .......... ......*... .......... .......... .......... *...*P.... ......*... ...
OR504670.1 This study         *......... .......... ......*... .......... .......... .......... *...*P.F.. ......*... ...
OR504671.1 This study         *......... .......... ......*... .......... .......... .......... *...*P.... ......*... ...
OR504672.1 This study         *......... .......... ......*... .......... .......... .......... *...*P.F.. ......*... ...
OR504673.1 This study         *......... .......... ......*... .......... .......... .......... *...*P.... ......*... ...
```

```
                                    280       290       300       310       320       330       340       350
                              ....|....| ....|....| ....|....| ....|....| ....|....| ....|....| ....|....| ....|....| ...
NC013590.2 (C-27)-Reference   *NVESDSHCL VYTSEXXANM CCGQRP*NSI PVXXGLDGPR TSCIXXRAYL EMPSXXNTMC *XFX*RTCLY XKILCG*RVH QRX
OL410296.1 Marial vaccine     *......... .......... ......*... .......... .......... .......... *...*..... ......*... ...
KR296657.1 Virbac vaccine     *......... .......... ......*... .......... .......... .......... *...*..... ......*... ...
KR381803.1 Intervet vaccine   *......... .......... ......*... .......... .......... .......... *...*..... ......*... ...
MH070348.1 (KANS-02)          *......... .......... ......*... .......... .......... .......... *...*..... ......*... ...
OR504673.1 This study         *......... .......... ......*... .......... .......... .......... *...*P.... ......*... ...
OR504674.1 This study         *......... .......... ......*... .......... .......... .......... *...*P.F.. ......*... ...
OR504675.1 This study         *......... .......... ......*... .......... .......... .......... *...*..... ......*... ...
OR504676.1 This study         *......... .......... ......*... .......... .......... .......... *...*P.... ......*... ...
OR504677.1 This study         *......... .......... ......*... .......... .......... .......... *...*P.F.. ......*... ...
OR504678.1 This study         *......... .......... ......*... .......... .......... .......... *...*..... ......*... ...
OR504679.1 This study         *......... .......... ......*... .......... .......... .......... *...*P.... ......*... ...
OR504680.1 This study         *......... .......... ......*... .......... .......... .......... *...*P.F.. ......*... ...
OR504681.1 This study         *......... .......... ......*... .......... .......... .......... *...*..... ......*... ...
OR504682.1 This study         *......... .......... ......*... .......... .......... .......... *...*P.... ......*... ...
OR504683.1 This study         *......... .......... ......*... .......... .......... .......... *...*P.F.. ......*... ...
```

```
                                          280        290        300        310        320        330        340        350
                                 ....|....| ....|....| ....|....| ....|....| ....|....| ....|....| ....|....| ....|....| ...
NC013590.2 (C-27)-Reference      *NVESDSHCL VYTSEXXANM CCGQRP*NSI PVXXGLDGPR TSCIXXRAYL EMPSXXNTMC *XFX*RTCLY XKILCG*RVH QRX
OL410296.1 Marial vaccine        *......... .......... ......*... .......... .......... .......... *...*..... ......*... ...
KR296657.1 Virbac vaccine        *......... .......... ......*... .......... .......... .......... *...*..... ......*... ...
KR381803.1 Intervet vaccine      *......... .......... ......*... .......... .......... .......... *...*..... ......*... ...
MH070348.1 (KANS-02)             *......... .......... ......*... .......... .......... .......... *...*..... ......*... ...
OR504684.1 This study            *......... .......... ......*... .......... .......... .......... *...*..... ......*... ...
OR504685.1 This study            *......... .......... ......*... .......... .......... .......... *...*P.... ......*... ...
OR504686.1 This study            *......... .......... ......*... .......... .......... .......... *...*P.F.. ......*... ...
OR504687.1 This study            *......... .......... ......*... .......... .......... .......... *...*P.... ......*... ...
OR504688.1 This study            *......... .......... ......*... .......... .......... .......... *...*P.F.. ......*... ...
OR504689.1 This study            *......... .......... ......*... .......... .......... .......... *...*P.... ......*... ...
OR504690.1 This study            *......... .......... ......*... .......... .......... .......... *...*P.F.. ......*... ...
OR504691.1 This study            *......... .......... ......*... .......... .......... .......... *...*..... ......*... ...
OR504692.1 This study            *......... .......... ......*... .......... .......... .......... *...*..... ......*... ...
OR504693.1 This study            *......... .......... ......*... .......... .......... .......... *...*..... ......*... ...
OR504694.1 This study            *......... .......... ......*... .......... .......... .......... *...*P.... ......*... ...
```

```
                                          280        290        300        310        320        330        340        350
                                 ....|....| ....|....| ....|....| ....|....| ....|....| ....|....| ....|....| ....|....| ...
NC013590.2 (C-27)-Reference      *NVESDSHCL VYTSEXXANM CCGQRP*NSI PVXXGLDGPR TSCIXXRAYL EMPSXXNTMC *XFX*RTCLY XKILCG*RVH QRX
OL410296.1 Marial vaccine        *......... .......... ......*... .......... .......... .......... *...*..... ......*... ...
KR296657.1 Virbac vaccine        *......... .......... ......*... .......... .......... .......... *...*..... ......*... ...
KR381803.1 Intervet vaccine      *......... .......... ......*... .......... .......... .......... *...*..... ......*... ...
MH070348.1 (KANS-02)             *......... .......... ......*... .......... .......... .......... *...*..... ......*... ...
OR504695.1 This study            *......... .......... ......*... .......... .......... .......... *...*P.F.. ......*... ...
OR504696.1 This study            *......... .......... ......*... .......... .......... .......... *...*..... ......*... ...
OR504697.1 This study            *......... .......... ......*... .......... .......... .......... *...*P.... ......*... ...
OR504698.1 This study            *......... .......... ......*... .......... .......... .......... *...*P.F.. ......*... ...
OR504699.1 This study            *......... .......... ......*... .......... .......... .......... *...*..... ......*... ...
OR504700.1 This study            *......... .......... ......*... .......... .......... .......... *...*P.... ......*... ...
OR504701.1 This study            *......... .......... ......*... .......... .......... .......... *...*P.F.. ......*... ...
OR504702.1 This study            *......... .......... ......*... .......... .......... .......... *...*..... ......*... ...
OR504703.1 This study            *......... .......... ......*... .......... .......... .......... *...*P.... ......*... ...
OR504704.1 This study            *......... .......... ......*... .......... .......... .......... *...*P.F.. ......*... ...
OR504705.1 This study            *......... .......... ......*... .......... .......... .......... *...*..... ......*... ...
```
