# Supplementary material for: First report of molecular epidemiology and phylogenetic characteristics of feline herpesvirus (FHV-1) from naturally infected cats in Kunshan, China
Source: Virol J. 2024 May 22;21:115. doi: 10.1186/s12985-024-02391-1 (PMC11112849; doi:10.1186/s12985-024-02391-1)
Supplement: Supplementary file 4 — Supplementary Material 4 [file 12985_2024_2391_MOESM4_ESM.pdf]

```
                                     10         20         30         40         50         60         70         80         90
                              ....|....| ....|....| ....|....| ....|....| ....|....| ....|....| ....|....| ....|....| ....|....|
NC013590.2 (C-27)-Reference   MASGTIPVQN EEIIKSQVNT VRIYIDGAYG IGKSLTAKYL VRADENRPGY TYYFPEPMLY WRSLFETDVV GGIYAVQDRK RRGELSAEDA
OL410296.1 Marial vaccine     .......... .......... .......... .......... .......... .......... .......... .......... ..........
KR296657.1 Virbac vaccine     .......... .......... .......... .......... .......... .......... .......... .......... ..........
KR381803.1 Intervet vaccine   .......... .......... .......... .......... .......... .......... .......... .......... ..........
MH070348.1 FHV-1 (KANS-02)    .......... .......... .......... .......... .......... .......... .......... .......... ..........
MH070330.1 FHV-1 (SANJ-01)    .......... .........I .......... .......... .......... .......... .......... .......... ..........
OR504620.1 This study         .......... .......... .......... .......... .......... .......... .......... .......... ..........
OR504621.1 This study         .......... .......... .......... .......... .......... .......... .......... .......... ..........
OR504622.1 This study         .......... .......... .....E.... .......... .......... .......... .G........ .......... ..........
OR504623.1 This study         .......... .......... .......... .......... .......... .......... .......... .......... ..........
OR504624.1 This study         .......... .......... .....E.... .......... .......... .......... .G........ .......... ..........
OR504625.1 This study         .......... .......... .......... .......... .......... .......... .......... .......... ..........
OR504626.1 This study         .......... .......... .......... .......... .......... .......... .......... .......... ..........
OR504627.1 This study         .......... .......... .....E.... .......... .......... .......... .G........ .......... ..........
OR504628.1 This study         .......... .......... .......... .......... .......... .......... .......... .......... ..........
OR504629.1 This study         .......... .......... .......... .......... .......... .......... .......... .......... ..........
OR504630.1 This study         .......... .......... .....E.... .......... .......... .......... .G........ .......... ..........
```

```
                                     10         20         30         40         50         60         70         80         90
                              ....|....| ....|....| ....|....| ....|....| ....|....| ....|....| ....|....| ....|....| ....|....|
NC013590.2 (C-27)-Reference   MASGTIPVQN EEIIKSQVNT VRIYIDGAYG IGKSLTAKYL VRADENRPGY TYYFPEPMLY WRSLFETDVV GGIYAVQDRK RRGELSAEDA
OL410296.1 Marial vaccine     .......... .......... .......... .......... .......... .......... .......... .......... ..........
KR296657.1 Virbac vaccine     .......... .......... .......... .......... .......... .......... .......... .......... ..........
KR381803.1 Intervet vaccine   .......... .......... .......... .......... .......... .......... .......... .......... ..........
MH070348.1 FHV-1 (KANS-02)    .......... .......... .......... .......... .......... .......... .......... .......... ..........
MH070330.1 FHV-1 (SANJ-01)    .......... .........I .......... .......... .......... .......... .......... .......... ..........
OR504631.1 This study         .......... .......... .....E.... .......... .......... .......... .G........ .......... ..........
OR504632.1 This study         .......... .......... .....E.... .......... .......... .......... .G........ .......... ..........
OR504633.1 This study         .......... .......... .....E.... .......... .......... .......... .G........ .......... ..........
OR504634.1 This study         .......... .......... .....E.... .......... .......... .......... .G........ .......... ..........
OR504635.1 This study         .......... .......... .....E.... .......... .......... .......... .G........ .......... ..........
OR504636.1 This study         .......... .......... .....E.... .......... .......... .......... .G........ .......... ..........
OR504637.1 This study         .......... .......... .....E.... .......... .......... .......... .G........ .......... ..........
OR504638.1 This study         .......... .......... .....E.... .......... .......... .......... .G........ .......... ..........
OR504639.1 This study         .......... .......... .....E.... .......... .......... .......... .G........ .......... ..........
OR504640.1 This study         .......... .......... .....E.... .......... .......... .......... .G........ .......... ..........
OR504641.1 This study         .......... .......... .....E.... .......... .......... .......... .G........ .......... ..........
```

```
                                       10         20         30         40         50         60         70         80         90
                                ....|....| ....|....| ....|....| ....|....| ....|....| ....|....| ....|....| ....|....| ....|....|
NC013590.2 (C-27)-Reference    MASGTIPVQN EEIIKSQVNT VRIYIDGAYG IGKSLTAKYL VRADENRPGY TYYFPEPMLY WRSLFETDVV GGIYAVQDRK RRGELSAEDA
OL410296.1 Marial vaccine      .......... .......... .......... .......... .......... .......... .......... .......... ..........
KR296657.1 Virbac vaccine      .......... .......... .......... .......... .......... .......... .......... .......... ..........
KR381803.1 Intervet vaccine    .......... .......... .......... .......... .......... .......... .......... .......... ..........
MH070348.1 FHV-1 (KANS-02)     .......... .......... .......... .......... .......... .......... .......... .......... ..........
MH070330.1 FHV-1 (SANJ-01)     .......... .........I .......... .......... .......... .......... .......... .......... ..........
OR504642.1 This study          .......... .......... .....E.... .......... .......... .......... .G........ .......... ..........
OR504643.1 This study          .......... .......... .......... .......... .......... .......... .......... .......... ..........
OR504644.1 This study          .......... .......... .......... .......... .......... .......... .......... .......... ..........
OR504645.1 This study          .......... .......... .......... .......... .......... .......... .......... .......... ..........
OR504646.1 This study          .......... .......... .......... .......... .......... .......... .......... .......... ..........
OR504647.1 This study          .......... .......... .......... .......... .......... .......... .......... .......... ..........
OR504648.1 This study          .......... .......... .......... .......... .......... .......... .......... .......... ..........
OR504649.1 This study          .......... .......... .......... .......... .......... .......... .......... .......... ..........
OR504650.1 This study          .......... .......... .......... .......... .......... .......... .......... .......... ..........
OR504651.1 This study          .......... .......... .......... .......... .......... .......... .......... .......... ..........
```

```
                                       10         20         30         40         50         60         70         80         90
                                ....|....| ....|....| ....|....| ....|....| ....|....| ....|....| ....|....| ....|....| ....|....|
NC013590.2 (C-27)-Reference    MASGTIPVQN EEIIKSQVNT VRIYIDGAYG IGKSLTAKYL VRADENRPGY TYYFPEPMLY WRSLFETDVV GGIYAVQDRK RRGELSAEDA
OL410296.1 Marial vaccine      .......... .......... .......... .......... .......... .......... .......... .......... ..........
KR296657.1 Virbac vaccine      .......... .......... .......... .......... .......... .......... .......... .......... ..........
KR381803.1 Intervet vaccine    .......... .......... .......... .......... .......... .......... .......... .......... ..........
MH070348.1 FHV-1 (KANS-02)     .......... .......... .......... .......... .......... .......... .......... .......... ..........
MH070330.1 FHV-1 (SANJ-01)     .......... .........I .......... .......... .......... .......... .......... .......... ..........
OR504652.1 This study          .......... .......... .......... .......... .......... .......... .......... .......... ..........
OR504653.1 This study          .......... .......... .....E.... .......... .......... .......... .G........ .......... ..........
OR504654.1 This study          .......... .......... .....E.... .......... .......... .......... .G........ .......... ..........
OR504655.1 This study          .......... .......... .....E.... .......... .......... .......... .G........ .......... ..........
OR504656.1 This study          .......... .......... .....E.... .......... .......... .......... .G........ .......... ..........
OR504657.1 This study          .......... .......... .....E.... .......... .......... .......... .G........ .......... ..........
OR504658.1 This study          .......... .......... .....E.... .......... .......... .......... .G........ .......... ..........
OR504659.1 This study          .......... .......... .....E.... .......... .......... .......... .G........ .......... ..........
OR504660.1 This study          .......... .......... .....E.... .......... .......... .......... .G........ .......... ..........
OR504661.1 This study          .......... .......... .....E.... .......... .......... .......... .G........ .......... ..........
OR504662.1 This study          .......... .......... .......... .......... .......... .......... .......... .......... ..........
```

```
                                            100        110        120        130        140        150        160        170        180
                                    ....|....| ....|....| ....|....| ....|....| ....|....| ....|....| ....|....| ....|....| ....|....|
NC013590.2 (C-27)-Reference         AYITAHYQAR FAAPYLLLHS RLSTITGYQK VVCEEHPDVT LIIDRHPLAS LVCFPLARYF VGDMTLGSVL SLMATLPREP PGGNLVVTTL
OL410296.1 Marial vaccine           .......... .......... .......... .......... .......... .......... .......... .......... ..........
KR296657.1 Virbac vaccine           .......... .......... .......... .......... .......... .......... .......... .......... ..........
KR381803.1 Intervet vaccine         .......... .......... .......... .......... .......... .......... .......... .......... ..........
MH070348.1 FHV-1 (KANS-02)          .......... .......... .......... .......... .......... .......... .......... .......... ..........
MH070330.1 FHV-1 (SANJ-01)          .......... .......... .......... .......... .......... .......... .......... .......... ..........
OR504620.1 This study               .......... .......... .......... .......... .......... .......... .......... .......... ..........
OR504621.1 This study               .......... .......... .......... .......... .......... .......... .......... .......... ..........
OR504622.1 This study               .......... .......... .......... .......... .......... .......... .......... .......... ..........
OR504623.1 This study               .......... .......... .......... .......... .......... .......... .......... .......... ..........
OR504624.1 This study               .......... .......... .......... .......... .......... .......... .......... .......... ..........
OR504625.1 This study               .......... .......... .......... .......... .......... .......... .......... .......... ..........
OR504626.1 This study               .......... .......... .......... .......... .......... .......... .......... .......... ..........
OR504627.1 This study               .......... .......... .......... .......... .......... .......... .......... .......... ..........
OR504628.1 This study               .......... .......... .......... .......... .......... .......... .......... .......... ..........
OR504629.1 This study               .......... .......... .......... .......... .......... .......... .......... .......... ..........
OR504630.1 This study               .......... .......... .......... .......... .......... .......... .......... .......... ..........
```

```
                                            100        110        120        130        140        150        160        170        180
                                    ....|....| ....|....| ....|....| ....|....| ....|....| ....|....| ....|....| ....|....| ....|....|
NC013590.2 (C-27)-Reference         AYITAHYQAR FAAPYLLLHS RLSTITGYQK VVCEEHPDVT LIIDRHPLAS LVCFPLARYF VGDMTLGSVL SLMATLPREP PGGNLVVTTL
OL410296.1 Marial vaccine           .......... .......... .......... .......... .......... .......... .......... .......... ..........
KR296657.1 Virbac vaccine           .......... .......... .......... .......... .......... .......... .......... .......... ..........
KR381803.1 Intervet vaccine         .......... .......... .......... .......... .......... .......... .......... .......... ..........
MH070348.1 FHV-1 (KANS-02)          .......... .......... .......... .......... .......... .......... .......... .......... ..........
MH070330.1 FHV-1 (SANJ-01)          .......... .......... .......... .......... .......... .......... .......... .......... ..........
OR504631.1 This study               .......... .......... .......... .......... .......... .......... .......... .......... ..........
OR504632.1 This study               .......... .......... .......... .......... .......... .......... .......... .......... ..........
OR504633.1 This study               .......... .......... .......... .......... .......... .......... .......... .......... ..........
OR504634.1 This study               .......... .......... .......... .......... .......... .......... .......... .......... ..........
OR504635.1 This study               .......... .......... .......... .......... .......... .......... .......... .......... ..........
OR504636.1 This study               .......... .......... .......... .......... .......... .......... .......... .......... ..........
OR504637.1 This study               .......... .......... .......... .......... .......... .......... .......... .......... ..........
OR504638.1 This study               .......... .......... .......... .......... .......... .......... .......... .......... ..........
OR504639.1 This study               .......... .......... .......... .......... .......... .......... .......... .......... ..........
OR504640.1 This study               .......... .......... .......... .......... .......... .......... .......... .......... ..........
OR504641.1 This study               .......... .......... .......... .......... .......... .......... .......... .......... ..........
```

```
                                     100       110       120       130       140       150       160       170       180
                              ....|....| ....|....| ....|....| ....|....| ....|....| ....|....| ....|....| ....|....| ....|....|
NC013590.2 (C-27)-Reference   AYITAHYQAR FAAPYLLLHS RLSTITGYQK VVCEEHPDVT LIIDRHPLAS LVCFPLARYF VGDMTLGSVL SLMATLPREP PGGNLVVTTL
OL410296.1 Marial vaccine     .......... .......... .......... .......... .......... .......... .......... .......... ..........
KR296657.1 Virbac vaccine     .......... .......... .......... .......... .......... .......... .......... .......... ..........
KR381803.1 Intervet vaccine   .......... .......... .......... .......... .......... .......... .......... .......... ..........
MH070348.1 FHV-1 (KANS-02)    .......... .......... .......... .......... .......... .......... .......... .......... ..........
MH070330.1 FHV-1 (SANJ-01)    .......... .......... .......... .......... .......... .......... .......... .......... ..........
OR504642.1 This study         .......... .......... .......... .......... .......... .......... .......... .......... ..........
OR504643.1 This study         .......... .......... .......... .......... .......... .......... .......... .......... ..........
OR504644.1 This study         .......... .......... .......... .......... .......... .......... .......... .......... ..........
OR504645.1 This study         .......... .......... .......... .......... .......... .......... .......... .......... ..........
OR504646.1 This study         .......... .......... .......... .......... .......... .......... .......... .......... ..........
OR504647.1 This study         .......... .......... .......... .......... .......... .......... .......... .......... ..........
OR504648.1 This study         .......... .......... .......... .......... .......... .......... .......... .......... ..........
OR504649.1 This study         .......... .......... .......... .......... .......... .......... .......... .......... ..........
OR504650.1 This study         .......... .......... .......... .......... .......... .......... .......... .......... ..........
OR504651.1 This study         .......... .......... .......... .......... .......... .......... .......... .......... ..........
```

```
                                     100       110       120       130       140       150       160       170       180
                              ....|....| ....|....| ....|....| ....|....| ....|....| ....|....| ....|....| ....|....| ....|....|
NC013590.2 (C-27)-Reference   AYITAHYQAR FAAPYLLLHS RLSTITGYQK VVCEEHPDVT LIIDRHPLAS LVCFPLARYF VGDMTLGSVL SLMATLPREP PGGNLVVTTL
OL410296.1 Marial vaccine     .......... .......... .......... .......... .......... .......... .......... .......... ..........
KR296657.1 Virbac vaccine     .......... .......... .......... .......... .......... .......... .......... .......... ..........
KR381803.1 Intervet vaccine   .......... .......... .......... .......... .......... .......... .......... .......... ..........
MH070348.1 FHV-1 (KANS-02)    .......... .......... .......... .......... .......... .......... .......... .......... ..........
MH070330.1 FHV-1 (SANJ-01)    .......... .......... .......... .......... .......... .......... .......... .......... ..........
OR504652.1 This study         .......... .......... .......... .......... .......... .......... .......... .......... ..........
OR504653.1 This study         .......... .......... .......... .......... .......... .......... .......... .......... ..........
OR504654.1 This study         .......... .......... .......... .......... .......... .......... .......... .......... ..........
OR504655.1 This study         .......... .......... .......... .......... .......... .......... .......... .......... ..........
OR504656.1 This study         .......... .......... .......... .......... .......... .......... .......... .......... ..........
OR504657.1 This study         .......... .......... .......... .......... .......... .......... .......... .......... ..........
OR504658.1 This study         .......... .......... .......... .......... .......... .......... .......... .......... ..........
OR504659.1 This study         .......... .......... .......... .......... .......... .......... .......... .......... ..........
OR504660.1 This study         .......... .......... .......... .......... .......... .......... .......... .......... ..........
OR504661.1 This study         .......... .......... .......... .......... .......... .......... .......... .......... ..........
OR504662.1 This study         .......... .......... .......... .......... .......... .......... .......... .......... ..........
```

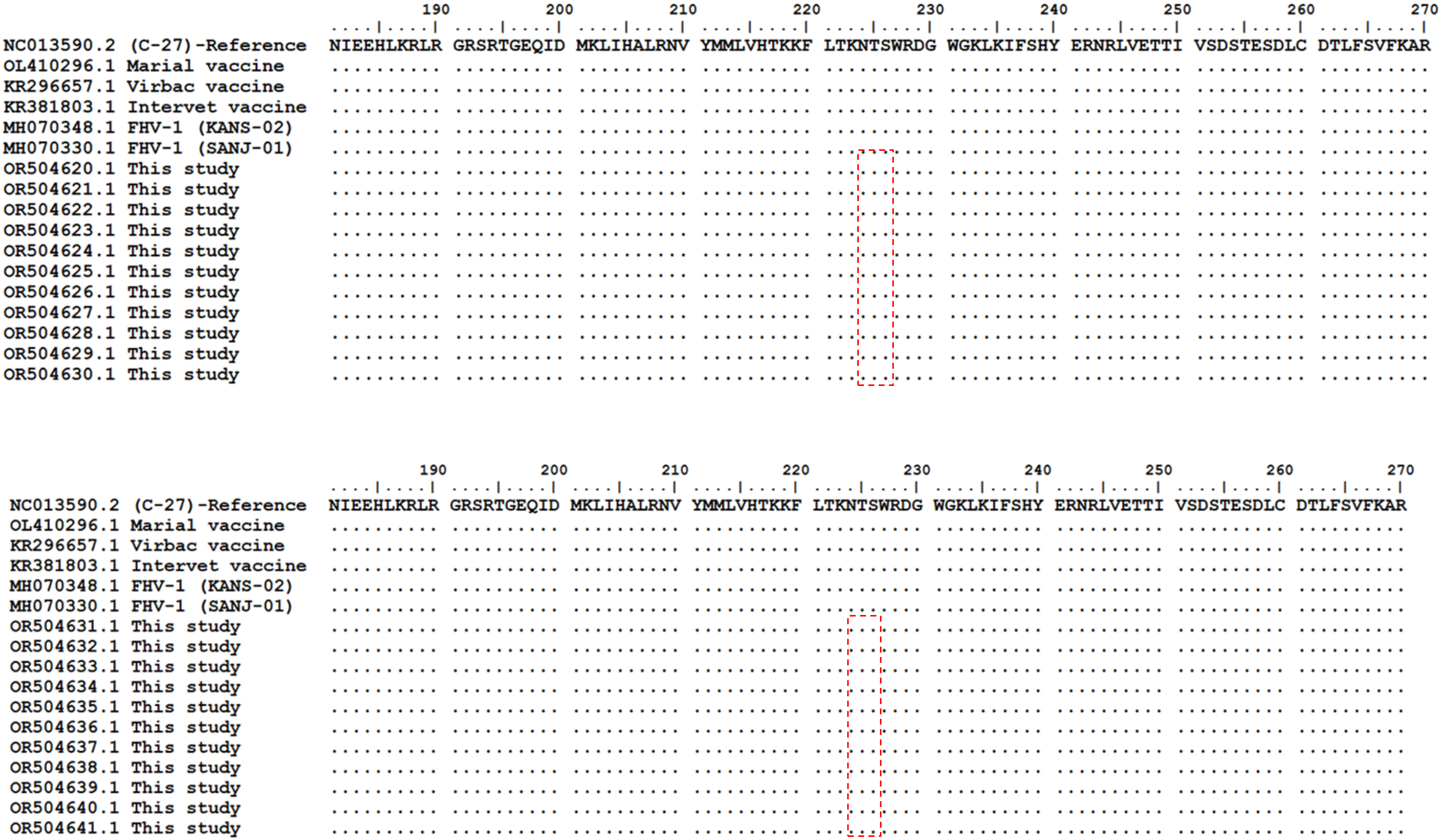
190 200 210 220 230 240 250 260 270
NC013590.2 (C-27)-Reference NIEEHLKRLR GRSRTGEQID MKLIHALRNV YMMLVHTKKF LTKNTSWRDG WGKLKIFSHY ERNRLVETTI VSDSTESDLC DTLFSVFKAR
OL410296.1 Marial vaccine
KR296657.1 Virbac vaccine
KR381803.1 Intervet vaccine
MH070348.1 FHV-1 (KANS-02)
MH070330.1 FHV-1 (SANJ-01)
OR504620.1 This study
OR504621.1 This study
OR504622.1 This study
OR504623.1 This study
OR504624.1 This study
OR504625.1 This study
OR504626.1 This study
OR504627.1 This study
OR504628.1 This study
OR504629.1 This study
OR504630.1 This study
190 200 210 220 230 240 250 260 270
NC013590.2 (C-27)-Reference NIEEHLKRLR GRSRTGEQID MKLIHALRNV YMMLVHTKKF LTKNTSWRDG WGKLKIFSHY ERNRLVETTI VSDSTESDLC DTLFSVFKAR
OL410296.1 Marial vaccine
KR296657.1 Virbac vaccine
KR381803.1 Intervet vaccine
MH070348.1 FHV-1 (KANS-02)
MH070330.1 FHV-1 (SANJ-01)
OR504631.1 This study
OR504632.1 This study
OR504633.1 This study
OR504634.1 This study
OR504635.1 This study
OR504636.1 This study
OR504637.1 This study
OR504638.1 This study
OR504639.1 This study
OR504640.1 This study
OR504641.1 This study

```
                                          190       200       210       220       230       240       250       260       270
                                   ....|....| ....|....| ....|....| ....|....| ....|....| ....|....| ....|....| ....|....| ....|....|
NC013590.2 (C-27)-Reference        NIEEHLKRLR GRSRTGEQID MKLIHALRNV YMMLVHTKKF LTKNTSWRDG WGKLKIFSHY ERNRLVETTI VSDSTESDLC DTLFSVFKAR
OL410296.1 Marial vaccine          .......... .......... .......... .......... .......... .......... .......... .......... ..........
KR296657.1 Virbac vaccine          .......... .......... .......... .......... .......... .......... .......... .......... ..........
KR381803.1 Intervet vaccine        .......... .......... .......... .......... .......... .......... .......... .......... ..........
MH070348.1 FHV-1 (KANS-02)         .......... .......... .......... .......... .......... .......... .......... .......... ..........
MH070330.1 FHV-1 (SANJ-01)         .......... .......... .......... .......... .......... .......... .......... .......... ..........
OR504642.1 This study              .......... .......... .......... .......... .......... .......... .......... .......... ..........
OR504643.1 This study              .......... .......... .......... .......... .......... .......... .......... .......... ..........
OR504644.1 This study              .......... .......... .......... .......... .......... .......... .......... .......... ..........
OR504645.1 This study              .......... .......... .......... .......... .......... .......... .......... .......... ..........
OR504646.1 This study              .......... .......... .......... .......... .......... .......... .......... .......... ..........
OR504647.1 This study              .......... .......... .......... .......... .......... .......... .......... .......... ..........
OR504648.1 This study              .......... .......... .......... .......... .......... .......... .......... .......... ..........
OR504649.1 This study              .......... .......... .......... .......... .......... .......... .......... .......... ..........
OR504650.1 This study              .......... .......... .......... .......... .......... .......... .......... .......... ..........
OR504651.1 This study              .......... .......... .......... .......... .......... .......... .......... .......... ..........

                                          190       200       210       220       230       240       250       260       270
                                   ....|....| ....|....| ....|....| ....|....| ....|....| ....|....| ....|....| ....|....| ....|....|
NC013590.2 (C-27)-Reference        NIEEHLKRLR GRSRTGEQID MKLIHALRNV YMMLVHTKKF LTKNTSWRDG WGKLKIFSHY ERNRLVETTI VSDSTESDLC DTLFSVFKAR
OL410296.1 Marial vaccine          .......... .......... .......... .......... .......... .......... .......... .......... ..........
KR296657.1 Virbac vaccine          .......... .......... .......... .......... .......... .......... .......... .......... ..........
KR381803.1 Intervet vaccine        .......... .......... .......... .......... .......... .......... .......... .......... ..........
MH070348.1 FHV-1 (KANS-02)         .......... .......... .......... .......... .......... .......... .......... .......... ..........
MH070330.1 FHV-1 (SANJ-01)         .......... .......... .......... .......... .......... .......... .......... .......... ..........
OR504652.1 This study              .......... .......... .......... .......... .......... .......... .......... .......... ..........
OR504653.1 This study              .......... .......... .......... .......... .......... .......... .......... .......... ..........
OR504654.1 This study              .......... .......... .......... .......... .......... .......... .......... .......... ..........
OR504655.1 This study              .......... .......... .......... .......... .......... .......... .......... .......... ..........
OR504656.1 This study              .......... .......... .......... .......... .......... .......... .......... .......... ..........
OR504657.1 This study              .......... .......... .......... .......... .......... .......... .......... .......... ..........
OR504658.1 This study              .......... .......... .......... .......... .......... .......... .......... .......... ..........
OR504659.1 This study              .......... .......... .......... .......... .......... .......... .......... .......... ..........
OR504660.1 This study              .......... .......... .......... .......... .......... .......... .......... .......... ..........
OR504661.1 This study              .......... .......... .......... .......... .......... .......... .......... .......... ..........
OR504662.1 This study              .......... .......... .......... .......... .......... .......... .......... .......... ..........
```

```
                                      280        290        300        310        320        330        340
                                ....|....| ....|....| ....|....| ....|....| ....|....| ....|....| ....|....| ...
NC013590.2 (C-27)-Reference     ELSDQNGDLL DMHAWVLDGL METLQNLQIF TLNLEGTPDE CAAALGALRQ DMDMTFIAAC DMHRISEALT IYH
OL410296.1 Marial vaccine       .......... .......... .......... .......... .......... .......... .......... ...
KR296657.1 Virbac vaccine       .......... .......... .......... .......... .......... .......... .......... ...
KR381803.1 Intervet vaccine     .......... .......... .......... .......... .......... .......... .......... ...
MH070348.1 FHV-1 (KANS-02)      .......... .......... .......... .......... .......... .......... .......... ...
MH070330.1 FHV-1 (SANJ-01)      .......... .......... .......... .......... .......... .......... .......... ...
OR504620.1 This study           .......... .......... .......... .......... .......... .......... .......... ...
OR504621.1 This study           .......... .......... .......... .......... .......... .......... .......... ...
OR504622.1 This study           .......... .......... .......... .......... .......... .......... .......... ...
OR504623.1 This study           .......... .......... .......... .......... .......... .......... .......... ...
OR504624.1 This study           .......... .......... .......... .......... .......... .......... .......... ...
OR504625.1 This study           .......... .......... .......... .......... .......... .......... .......... ...
OR504626.1 This study           .......... .......... .......... .......... .......... .......... .......... ...
OR504627.1 This study           .......... .......... .......... .......... .......... .......... .......... ...
OR504628.1 This study           .......... .......... .......... .......... .......... .......... .......... ...
OR504629.1 This study           .......... .......... .......... .......... .......... .......... .......... ...
OR504630.1 This study           .......... .......... .......... .......... .......... .......... .......... ...
```

```
                                      280        290        300        310        320        330        340
                                ....|....| ....|....| ....|....| ....|....| ....|....| ....|....| ....|....| ...
NC013590.2 (C-27)-Reference     ELSDQNGDLL DMHAWVLDGL METLQNLQIF TLNLEGTPDE CAAALGALRQ DMDMTFIAAC DMHRISEALT IYH
OL410296.1 Marial vaccine       .......... .......... .......... .......... .......... .......... .......... ...
KR296657.1 Virbac vaccine       .......... .......... .......... .......... .......... .......... .......... ...
KR381803.1 Intervet vaccine     .......... .......... .......... .......... .......... .......... .......... ...
MH070348.1 FHV-1 (KANS-02)      .......... .......... .......... .......... .......... .......... .......... ...
MH070330.1 FHV-1 (SANJ-01)      .......... .......... .......... .......... .......... .......... .......... ...
OR504631.1 This study           .......... .......... .......... .......... .......... .......... .......... ...
OR504632.1 This study           .......... .......... .......... .......... .......... .......... .......... ...
OR504633.1 This study           .......... .......... .......... .......... .......... .......... .......... ...
OR504634.1 This study           .......... .......... .......... .......... .......... .......... .......... ...
OR504635.1 This study           .......... .......... .......... .......... .......... .......... .......... ...
OR504636.1 This study           .......... .......... .......... .......... .......... .......... .......... ...
OR504637.1 This study           .......... .......... .......... .......... .......... .......... .......... ...
OR504638.1 This study           .......... .......... .......... .......... .......... .......... .......... ...
OR504639.1 This study           .......... .......... .......... .......... .......... .......... .......... ...
OR504640.1 This study           .......... .......... .......... .......... .......... .......... .......... ...
OR504641.1 This study           .......... .......... .......... .......... .......... .......... .......... ...
```

```
                                        280       290       300       310       320       330       340
                                ....|....| ....|....| ....|....| ....|....| ....|....| ....|....| ....|....| ...
NC013590.2 (C-27)-Reference     ELSDQNGDLL DMHAWVLDGL METLQNLQIF TLNLEGTPDE CAAALGALRQ DMDMTFIAAC DMHRISEALT IYH
OL410296.1 Marial vaccine       .......... .......... .......... .......... .......... .......... .......... ...
KR296657.1 Virbac vaccine       .......... .......... .......... .......... .......... .......... .......... ...
KR381803.1 Intervet vaccine     .......... .......... .......... .......... .......... .......... .......... ...
MH070348.1 FHV-1 (KANS-02)      .......... .......... .......... .......... .......... .......... .......... ...
MH070330.1 FHV-1 (SANJ-01)      .......... .......... .......... .......... .......... .......... .......... ...
OR504642.1 This study           .......... .......... .......... .......... .......... .......... .......... ...
OR504643.1 This study           .......... .......... .......... .......... .......... .......... .......... ...
OR504644.1 This study           .......... .......... .......... .......... .......... .......... .......... ...
OR504645.1 This study           .......... .......... .......... .......... .......... .......... .......... ...
OR504646.1 This study           .......... .......... .......... .......... .......... .......... .......... ...
OR504647.1 This study           .......... .......... .......... .......... .......... .......... .......... ...
OR504648.1 This study           .......... .......... .......... .......... .......... .......... .......... ...
OR504649.1 This study           .......... .......... .......... .......... .......... .......... .......... ...
OR504650.1 This study           .......... .......... .......... .......... .......... .......... .......... ...
OR504651.1 This study           .......... .......... .......... .......... .......... .......... .......... ...

                                ....|....| ....|....| ....|....| ....|....| ....|....| ....|....| ....|....| ...
NC013590.2 (C-27)-Reference     ELSDQNGDLL DMHAWVLDGL METLQNLQIF TLNLEGTPDE CAAALGALRQ DMDMTFIAAC DMHRISEALT IYH
OL410296.1 Marial vaccine       .......... .......... .......... .......... .......... .......... .......... ...
KR296657.1 Virbac vaccine       .......... .......... .......... .......... .......... .......... .......... ...
KR381803.1 Intervet vaccine     .......... .......... .......... .......... .......... .......... .......... ...
MH070348.1 FHV-1 (KANS-02)      .......... .......... .......... .......... .......... .......... .......... ...
MH070330.1 FHV-1 (SANJ-01)      .......... .......... .......... .......... .......... .......... .......... ...
OR504652.1 This study           .......... .......... .......... .......... .......... .......... .......... ...
OR504653.1 This study           .......... .......... .......... .......... .......... .......... .......... ...
OR504654.1 This study           .......... .......... .......... .......... .......... .......... .......... ...
OR504655.1 This study           .......... .......... .......... .......... .......... .......... .......... ...
OR504656.1 This study           .......... .......... .......... .......... .......... .......... .......... ...
OR504657.1 This study           .......... .......... .......... .......... .......... .......... .......... ...
OR504658.1 This study           .......... .......... .......... .......... .......... .......... .......... ...
OR504659.1 This study           .......... .......... .......... .......... .......... .......... .......... ...
OR504660.1 This study           .......... .......... .......... .......... .......... .......... .......... ...
OR504661.1 This study           .......... .......... .......... .......... .......... .......... .......... ...
OR504662.1 This study           .......... .......... .......... .......... .......... .......... .......... ...
```
