# Supplementary material for: First report of molecular epidemiology and phylogenetic characteristics of feline herpesvirus (FHV-1) from naturally infected cats in Kunshan, China
Source: Virol J. 2024 May 22;21:115. doi: 10.1186/s12985-024-02391-1 (PMC11112849; doi:10.1186/s12985-024-02391-1)
Supplement: Supplementary file 5 — Supplementary Material 5 [file 12985_2024_2391_MOESM5_ESM.pdf]

Gel photos are not available
